# Pharmacogenetic association study of cannabis use in chronic pain

**DOI:** 10.1186/s42238-026-00408-w

**Published:** 2026-02-14

**Authors:** William Beauchesne, Jordan Turcotte, Philippe Mercier, Flore Lavoie, Laurence Tessier, Ann-Lorie Gagnon, Catherine Allard, Elliot Fortin, Guillaume Léonard, Louis Gendron, Karine Tremblay

**Affiliations:** 1 Pharmacology-Physiology Department, Université de Sherbrooke, Saguenay, QC Canada; 2 Centre de recherche et d’innovation, Centre Intégré Universitaire de Santé et de Services Sociaux (CIUSSS) du Saguenay-Lac-Saint-Jean, Saguenay, Québec Canada; 3 Biochemistry and Functional Genomic Department, Université de Sherbrooke, Saguenay, QC Canada; 4 School of Rehabilitation, Faculté de Médecine et des Sciences de la Santé, Université de Sherbrooke, Sherbrooke, QC Canada; 5 Research Center on Aging, CIUSSS de L’Estrie-CHUS, Sherbrooke, QC Canada; 6 Institut de Pharmacologie de Sherbrooke, Université de Sherbrooke, Sherbrooke, QC Canada; 7 Centre de Recherche du Centre Hospitalier Universitaire de Sherbrooke (CRCHUS), Sherbrooke, QC Canada; 8 Quebec Pain Research Network, Sherbrooke, QC Canada

**Keywords:** Cannabis, Chronic pain, Pharmacogenetics, Psychosis, Cannabis use disorder

## Abstract

**Background:**

Pain is one of the leading causes of disability worldwide. Despite the various pharmacological treatments available, patients with chronic pain often remain with significant disabilities and unsatisfactory pain control. Cannabis and cannabinoids are sometimes used in the treatment of chronic pain as they have been shown to be useful in a subset of patients. Some of the adverse effects associated with cannabis use, such as cannabis use disorder (CUD) and cannabis-induced psychosis, have been associated with several genetic variants. Despite this, the paucity of the data or the contradictory results for reported variants limits our ability to use them as genetic markers to personalize cannabis treatment tailored to patients’ genetic background. The aim of this genetic association study was to investigate the link between previously reported genes and cannabinoid response in terms of pain response, CUD and risk of psychotic adverse events in patients with chronic pain.

**Methods:**

Phone or in person interviews were conducted to document participants’ characteristics, cannabis use and effects, concurrent pharmacotherapy and comorbid conditions. Screening for CUD was performed using the Cannabis Use Disorders Identification Test – Revised. Blood or saliva samples were collected for the genotyping of 18 variants in 11 genes (*BDNF*, *CNR1*, *CNR2*, *COMT*, *CYP2C9*, *FAAH*, *GABRA2*, *HES7*, *KAT2B*, *NRG1* and *OPMR1*).

**Results:**

One hundred participants were recruited, with blood or saliva samples collected from 77 of them. Two single-nucleotide polymorphisms (SNP) in cannabinoid receptor 1 (*CNR1*) were associated, before multiple testing correction, with psychotic adverse events. Namely, T allele carriage of the *CNR1* rs1049353 C > T variant increased the odds of having psychotic adverse events (OR = 6.1, 95% CI 1.7 – 27.9, *p*-value = 0,009) and C allele carriage of the *CNR1* rs2023239 T > C intronic variant also increased these odds (OR = 3.5, 95% CI 1.5 – 9.4, *p*-value = 0,033). These findings were not significant after adjustment for multiple SNPs testing and none of the variants were associated with CUD or pain response.

**Conclusions:**

These results suggest alternative allele carriers of rs1049353 and rs2023239 could be at an increased risk of psychotic adverse events related to cannabis use, although additional investigation is required to replicate and confirm these findings.

**Supplementary Information:**

The online version contains supplementary material available at 10.1186/s42238-026-00408-w.

## Background

Chronic pain ranks among the top causes of disability-adjusted life years in the world (GBD 2016 Disease and Injury Incidence and Prevalence Collaborators 2017). In Canada, almost one in five adults (7.6 million) lives with such pain (Santé Canada 2021). Despite its high prevalence and substantial impact on patient lives, the management of chronic pain remains particularly challenging (Cohen et al. 2021). Access to cannabis for medical purposes, such as in the treatment of chronic pain, has been available in Canada since 2001, while non-medical use of cannabis was legalized in 2018. A recent post-legalization study reported that 30.1% of adults living with chronic pain had used cannabis in the past year in the management of their condition (Godbout-Parent et al. 2022).

While the evidence regarding the efficacy of cannabis and cannabinoids in the treatment of chronic pain is limited, the latest meta-analysis has demonstrated significant – albeit small to very small – improvements in pain response among patients with chronic pain (Wang et al. 2021). However, there remains a significant proportion of patients (up to 70%) who do not achieve adequate pain response and no factor has reliably been identified as a predictor of this response (Poli et al. 2022; Allan et al. 2018).

Even though cannabis use is relatively widespread and is becoming more accessible (Government of Canada PS and PC 2002), either for self-management or with the help of a healthcare professional, it is well documented that its use is often discontinued due to adverse events (Volkow et al. 2014; Allan et al. 2018). Two significant adverse events, cannabis use disorder which is characterized by impaired control over cannabis use (Foll et al. 2026) and psychotic adverse events such as hallucinations or delusions (Schoeler et al. 2024), have been associated with multiple genetic variants (Hryhorowicz et al. 2018; Carvalho and Vieira-Coelho 2022). These variants could ultimately be used as genetic markers to personalize cannabis treatment and offer treatment tailored to the genetic background of patients, thereby reducing the potential harms when cannabis is used. Genetic variants could also be employed to identify patients who are more likely to benefit from cannabis prior to treatment initiation. Despite this, the paucity of the data on some of the previously reported variants and inconsistent results regarding some of them limit our ability to use them as genetic markers at the moment (Hryhorowicz et al. 2018; Babayeva and Loewy 2023).

Recent articles proposing frameworks of cannabis use in pain management illustrate the increased interest in the underlying genetic variation to pain response and could lead to a more appropriate use of these molecules (Visibelli et al. 2023; Visibelli et al. 2025; Kalak et al. 2025). Some even propose machine learning assisted methods but note the need to continue studying predictive markers of response, including genetic variants, to strengthen clinical algorithms (Visibelli et al. 2023; Visibelli et al. 2025). A recent review by Kalak et al*.* proposes a clinical guideline for cannabis use and arrives at the same conclusion: while some variants have a well-established impact on cannabis response, most variants warrant more research before they can be considered reliable clinical decision tools (Kalak et al. 2025). For example, in the endocannabinoid system, they describe the significant role of AKT Serine/Threonine Kinase 1 (*AKT1*) in the presence of psychotic symptoms while Catechol-O-Methyltransferase (*COMT*) did not show a significant enough effect to be considered for routine use in their review of observational studies.

The aim of this retrospective case–control genetic association study is to investigate the effect of different genetic variants, by a candidate-gene approach, on treatment response phenotypes in past or current users of cannabis or cannabinoids in their chronic pain treatment. This paper presents the relationship between three main response phenotypes (i.e., pain response, CUD and psychotic adverse events) and 28 genetic variants located in 17 genes selected for their previous association to response phenotype or their potential influence on cannabis’ pharmacokinetic or pharmacodynamic (Supplementary Table S1).

## Methods

### Study population and inclusion criteria

**Fig. 1:**
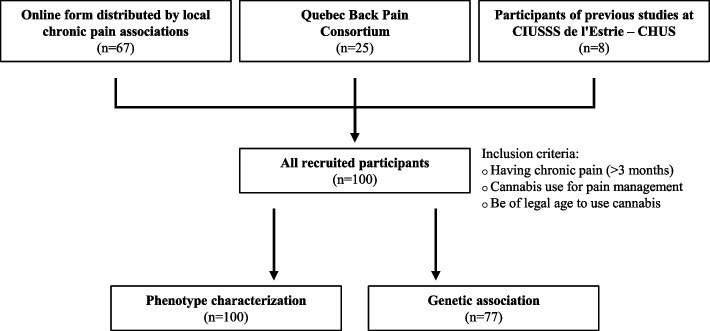
Flow chart illustrating recruitment methods and number of participants included in the analyses. In total, 100 participants were recruited in our study. Methods of recruitment included an online form distributed by local chronic pain association (*n =* 67), the Quebec Back Pain Consortium (*n =* 25) and a list of participants previously included in studies at CIUSSS de l’Estrie – CHUS (*n =* 8). All participants were included in phenotype characterization (*n =* 100) and participants with DNA samples were included in genetic association analyses (*n =* 77)

This multicentric retrospective genetic association study was conducted at the “Centre intégré universitaire de santé et de services sociaux du Saguenay—Lac-Saint-Jean” (CIUSSS-SLSJ) and “Centre intégré universitaire de santé et de services sociaux de l’Estrie – Centre hospitalier universitaire de Sherbrooke” (CIUSSS de l’Estrie – CHUS), two University Hospitals in Quebec, Canada. In total, 100 participants were recruited (Fig. 1). The majority of participants (67%) were recruited with an online form distributed by local chronic pain associations, either via social media or online advertisements. The remaining participants were recruited from the Quebec Back Pain Consortium (25%) and from participant lists included in previous studies conducted at CIUSSS de l’Estrie – CHUS (8%).

Inclusion criteria were self-reported and included: 1) having chronic pain (pain lasting longer than 3 months); 2) using or having used cannabis as a means to reduce pain associated with a chronic pain condition (either prescribed by a physician or in the context of self-management); 3) to be of legal age to use cannabis according to Quebec regulations at the time of the study (≥ 18 years old if prescribed by a physician and ≥ 21 years old if used in self-treatment). Participants were excluded if they self-reported never using cannabis for chronic pain relief. All recruited participants were included in phenotype characterization (*n =* 100), and only participants with available DNA samples were included in genetic association analyses (*n =* 77).

### Data collection

#### Timeline

Recruitment was conducted from October 2020 to July 2021. After obtaining free and informed consent, participants completed a primary survey either via telephone or during an in-person visit at one of the participating research centres. This first survey collected data on demographic characteristics, cannabis use, health status, medical history, and current pharmacologic therapies. Subsequently, biological samples – either blood (~ 10 ml) or saliva (~ 4 ml) – were collected on participant preference for DNA extraction. Participants were then invited to complete an online follow-up survey during a subsequent episode of cannabis use to evaluate its effect on pain. This assessment was made using the numerical pain rating scale (NRS) from 0 to 10 (“no pain” to “worst pain imaginable”) (Breivik et al. 2008; Ferreira-Valente et al. 2011) before and after cannabis use.

#### Assessments

Demographics included age, sex, life habits (tobacco use, alcohol use, and drugs), exercise, anthropometry and perception of their health using the European Quality of Life 5 Dimensions 5 Levels (EQ-5D-5L) instrument all collected in the primary survey (Herdman et al. 2011). The EQ-5D-5L instrument is a descriptive system looking at 5 dimensions of health (mobility, self-care, usual activities, pain/discomfort and anxiety/depression) in which each dimension has 5 possible levels (presenting no problem up to extreme problems) (Herdman et al. 2011). Health Index scores, which represents a combined score for the 5 dimensions and levels of health assessed by the EQ-5D-5L instrument, were calculated using the Canadian value set of the EQ-5D-5L (Xie et al. 2016).

Characteristics on participants’ cannabis use (e.g., age at first use, duration of use, frequency of use, routes of administration, quantities, delta9-tetrahydrocannabinol [THC] and cannabidiol [CBD] content of the products used) were also thoroughly assessed using an in-house questionnaire in the primary survey. Pain characteristics (e.g., pain intensity, impact of pain on physical function, neuropathic component) were documented using the Brief Pain Inventory (BPI) and “Douleur Neuropathique 4” (DN4) questionnaire on neuropathic pain (Bouhassira et al. 2005; Cleeland and Ryan 1994). Current cannabis use was defined as cannabis used in the past 6 months.

Three main phenotypes were assessed during this study and measured as binary outcomes: pain response, CUD and psychotic adverse events.

The online follow-up survey for pain response evaluation was composed of questions regarding the presence of somnolence or pain before and after their cannabis use. These elements were first evaluated before the consumption event and were reassessed 30 min to 4 h after use, at the onset of maximum effect according to the participants. The effect of cannabis on pain was assessed using the NRS from 0 to 10 (Breivik et al. 2008; Ferreira-Valente et al. 2011), and somnolence was assessed using the French version of the Stanford Sleepiness Scale (SSS) (Schulz et al. 1983). To assess if a participant had a positive response to cannabis with regards to pain response, they were asked to rate their average pain response with a percentage improvement in pain they typically experienced on a 0–100 (“no pain response” to “complete pain response”) numerical rating scale. With the online survey, adequate pain response was defined as a reduction of two points or ≥ 30% reduction of pain based on the NRS values before and after cannabis use. Data from the online survey were then used to assess the validity of the adequate pain response phenotype using the main questionnaire.

Screening for the presence of CUD was performed using the Cannabis Use Disorder Identification Test – Revised (CUDIT-R) (Bonn-Miller et al. 2016). The CUDIT-R requires participants to answer 8 multiple-choice questions about their cannabis use, which can be translated to a global score to assess the presence of CUD (Bonn-Miller et al. 2016). Scores ≥ 13 points were considered as indicating the presence of CUD.

Psychotic adverse events, collected in the primary survey, included the presence of hallucinations (visual, auditory or tactile) or delusions, and participants were classified as having had a psychotic adverse event if they had experienced at least one of those adverse reactions.

Study data was collected and managed using REDCap electronic data capture tools hosted at Université de Sherbrooke (Harris et al. 2019; Harris et al. 2009).

### SNP selection and genotyping

Genetic variants (single-nucleotide polymorphism, SNP) in candidate genes were identified through a literature review using PubMed database and ClinPGx (Whirl-Carrillo et al. 2012). SNPs reported in the literature associated at least once with either response to cannabis (e.g., psychiatric adverse events or CUD) or that could have an impact on the pharmacokinetics or pharmacodynamics of cannabis were selected for the study. This literature review identified 28 variants in 17 genes (ATP Binding Cassette Subfamily B Member 1 (*ABCB1*), *AKT1*, Brain Derived Neurotrophic Factor (*BDNF*), Cholinergic Receptor Muscarinic 3 (*CHRM3*), Cholinergic Receptor Nicotinic Alpha 2 Subunit (*CHRNA2*), Cannabinoid receptor 1(*CNR1*), Cannabinoid receptor 2 (*CNR2*), *COMT*, Cytochrome P450 Family 2 Subfamily C Member 9 (*CYP2C9*), Cytochrome P450 Family 3 Subfamily A Member 5 (*CYP3A5*), Cytochrome P450 Family 3 Subfamily A Member 5 (*FAAH*), Gamma-Aminobutyric Acid Type A Receptor Subunit Alpha2 (*GABRA2*), Hes Family BHLH Transcription Factor 7 (*HES7*), Lysine Acetyltransferase 2B (*KAT2B*), Neuregulin 1 (*NRG1*), Opioid Receptor Mu 1 (*OPRM1*), Purinergic Receptor P2X 7 (*P2RX7*), Supplementary Table S1).

Blood samples were collected in EDTA tubes, and the buffy coat was isolated in the 24 h following specimen collection. DNA extraction of buffy coat was performed using the Puregene Blood Kit (QIAGEN, Germany) and following the manufacturer’s procedure (QIAGEN n.d.). DNA extraction from saliva samples was done using the prepIT-L2P extraction kit (DNAgenoteck, Ottawa, Canada) directly from the sample we received by postal mail from participants using GenoTech® saliva sample collection kit OG-500 (DNAgenoteck, Ottawa, Canada) and following manufacturer’s protocol (DNAgentotek n.d.).

DNA samples were genotyped by standard TaqMan® method (Holland et al. 1991) at the Université de Sherbrooke RNomics platform lab. Details on genotyping, including the probe and primer designs used can be found in the Supplementary Table S2.

### Statistical analysis

Hardy–Weinberg Equilibrium (HWE) was tested for each variant. Assessment of the validity of genotyping was made based on HWE results following Holm-Bonferroni correction for multiple testing, genotyping call rate and minor allele frequency (MAF). Variants were excluded from subsequent analysis using the following criteria: 1) genotyping call rate inferior to 95%; 2) statistically significant departure from HWE (after multiple testing correction); 3) MAF inferior to 5%; 4) more than one alternative allele observed.

Categorical variables were compared using the Chi-square or Fisher’s exact tests (if > 20% of cells had expected frequencies < 5 or if a cell had an expected frequency of < 1). Normality of data was assessed by the Shapiro–Wilk Test. Comparisons between groups for continuous variables were made using independent samples t-test or Wilcoxon rank sum test (if the variable had a non-normal distribution).

Statistical tests were performed for each variant to identify potential statistical association with the three phenotypes assessed. Univariable logistic regression analyses using an additive genetic model were performed for variants with statistically significant associations with the studied phenotypes before multiple testing correction. Multiple testing corrections were performed according to the method proposed by Li, J. & Ji, L. (2005) for adjusting multilocus analyses by calculating the effective number of variants analyzed (Li and Ji 2005). Specifically, Bonferroni correction for genetic analyses was conducted for an effective number of 15 variants. Statistical significance threshold was set at *p* < 0.05 after correction. All analyses were performed using R Statistical Software (v4.2.1) (R Core Team 2023).

## Results

### Participants’ description

A total of 100 participants were included in the present study, and the characteristics of the studied sample are presented in Table 1. Participants were aged between 22 and 77 years old, and 67% were females. Most participants were current cannabis users at the time of the study (92%).

**Table 1:** Participant characteristics

	**Overall** (*N =* 100)	**Current use** (*N =* 92)	**Past use** (*N =* 8)	***p*-value**^*1*^
Demographics				
Female sex, n (%)	67 (67.0%)	61 (66.3%)	6 (75.0%)	> 0.99
Mean age in years (SD)	48.0 (13.1)	47.2 (12.7)	57.5 (14.7)	0.053
Ethnicity, *n* (%)^2^				
European	95 (95.9%)	87 (95.6%)	8 (100.0%)	> 0.99
Latino	2 (2.0%)	2 (2.2%)	0 (0.0%)	
Other	2 (2.0%)	2 (2.2%)	0 (0.0%)	
Chronic pain and health status				
DN4 score (≥ 4), *n* (%)	65 (65.0%)	59 (64.1%)	6 (75.0%)	0.71
Median pain duration in years (IQR)^3^	12.0 (7.0, 21.8)	12.0 (7.0, 21.8)	15.0 (4.5, 22.0)	0.82
Median BPI pain severity (IQR)^*2*^	5.25 (3.50, 6.12)	5.25 (3.50, 6.25)	5.25 (3.38, 6.00)	0.75
Median BPI pain interference (IQR)^4^	3.88 (1.67, 5.50)	3.88 (2.00, 5.62)	1.65 (0.67, 2.94)	**0.045**
Median EQ-5D-5L index (IQR)^*2*^	0.70 (0.50, 0.82)	0.68 (0.47, 0.81)	0.78 (0.67, 0.83)	0.15
Mean EQ VAS (SD)	61.8 (20.9)	60.9 (21.3)	72.5 (13.9)	0.13
Mean BMI in kg/m2 (SD)^3^	27.8 (6.2)	27.5 (6.0)	30.7 (7.5)	0.27
Cannabis use characteristics				
Main method of use, *n* (%)				0.10
Inhaled	44 (44.0%)	43 (46.7%)	1 (12.5%)	
Oral	45 (45.0%)	40 (43.5%)	5 (62.5%)	
Other or more than one	11 (11.0%)	9 (9.8%)	2 (25.0%)	
Frequency of use, *n* (%)				0.25
≤ Weekly	4 (4.0%)	3 (3.3%)	1 (12.5%)	
More than once per week	15 (15.0%)	13 (14.1%)	2 (25.0%)	
Daily	27 (27.0%)	26 (28.3%)	1 (12.5%)	
More than once daily	54 (54.0%)	50 (54.3%)	4 (50.0%)	
Mean age at first cannabis use in years (SD)	23.7 (15.4)	23.4 (15.0)	27.2 (20.6)	0.57
Past medical history, *n* (%)				
Musculoskeletal	97 (97.0%)	89 (96.7%)	8 (100.0%)	> 0.99
Psychiatric	70 (70.0%)	66 (71.7%)	4 (50.0%)	0.24
Gastrointestinal	53 (53.0%)	50 (54.3%)	3 (37.5%)	0.47
Neurologic	40 (40.0%)	36 (39.1%)	4 (50.0%)	0.71
Cardiovascular	39 (39.0%)	36 (39.1%)	3 (37.5%)	> 0.99
Respiratory	36 (36.0%)	34 (37.0%)	2 (25.0%)	0.71
Metabolic	26 (26.0%)	24 (26.1%)	2 (25.0%)	> 0.99
Cancer	10 (10.0%)	9 (9.8%)	1 (12.5%)	0.58
Concurrent pharmacotherapy, *n* (%)				
Antidepressants	55 (55.0%)	50 (54.3%)	5 (62.5%)	0.73
Acetaminophen	33 (33.0%)	31 (33.7%)	2 (25.0%)	> 0.99
NSAIDs	32 (32.0%)	29 (31.5%)	3 (37.5%)	0.71
Opioids	32 (32.0%)	28 (30.4%)	4 (50.0%)	0.26
Antiepileptics	28 (28.0%)	27 (29.3%)	1 (12.5%)	0.44
Muscle relaxants	19 (19.0%)	17 (18.5%)	2 (25.0%)	0.64
Benzodiazepines	15 (15.0%)	12 (13.0%)	3 (37.5%)	0.10
Stimulants	9 (9.0%)	9 (9.8%)	0 (0.0%)	> 0.99
Z drugs/benzodiazepine like	7 (7.0%)	7 (7.6%)	0 (0.0%)	> 0.99
Biologics/DMARDs	3 (3.0%)	2 (2.2%)	1 (12.5%)	0.22
Pain management using cannabis only	8 (8.0%)	7 (7.6%)	1 (12.5%)	0.50
Phenotypes, *n* (%)				
Pain response (≥ 30%)^*5*^	74 (78.7%)	72 (82.8%)	2 (28.6%)	**0.004**
Psychotic adverse events	6 (6.0%)	6 (6.5%)	0 (0.0%)	> 0.99
CUDIT-R ≥ 13	25 (25.0%)	25 (27.2%)	0 (0.0%)	0.20

Statistically significant *p*-values are bolded

*Abbreviations*: *DMARDs* Disease-modifying antirheumatic drugs, *NSAIDs* Non Steroidal Anti-Inflammatory Drugs

^1^Fisher’s exact test; Wilcoxon rank sum test

^2^Data available *N =* 99

^3^Data available *N =* 98

^4^Data available *N =* 97

^5^Data available *N =* 94

Among the health conditions and comorbidities of the participants, musculoskeletal disorders were the most common, present in almost all participants (97%). Psychiatric and gastrointestinal comorbidities were also frequent, being present in more than half of the participants (70% and 53%, respectively). The most frequent chronic pain-related diagnoses were back pain (69%), fibromyalgia (45%) and osteoarthritis (34%). Neuropathic pain was present in almost two thirds of the participants (65%). Patients reported having chronic pain for a median duration of 12.0 years (interquartile range (IQR) = 7.0–21.8 years) with an average severity of moderate pain (BPI pain score median (IQR) = 5.25 (3.50, 6.12)) and mild interference with daily life (BPI interference score median (IQR) = 3.88 (1.67, 5.50)).

The main methods of consumption used by the participants were oral (45%) and inhalation (44%). Among participants with inhaled use, the average quantity of inhaled cannabis was 1.22 g per day of use (SD = 1.05). Among those with oral THC use, median quantity ingested per day of use was 2.99 mg (IQR 1.07–9.50) while for CBD use, the median ingested quantity per day of use was 21.55 mg (IQR 12.40–48.15 mg). Daily use was frequent, with 81% using cannabis at least once per day. Most users with inhalation as their main method of use did so using cannabis with products containing at least twice the amount of THC compared to CBD (71%). The opposite was observed for participants consuming cannabis orally. Indeed, these participants were using products containing at least twice the amount of CBD compared to THC (69%). However, the information regarding THC and CBD content was missing for many participants.

The most frequent concurrent pharmacological treatments were antidepressants (55%) followed by acetaminophen (33%), nonsteroidal anti-inflammatory drugs (NSAIDs) (32%) and opioids (32%). Pain management using cannabis only, without any other concurrent pharmacological treatment, was done by 8% of participants.

No statistically significant differences were observed between past and current users concerning demographic characteristics, health status, past medical history or concurrent pharmacotherapy. However, participants with current cannabis use reported higher BPI interference score with a median value of 3.88 (IQR 2.00–5.62) compared to past users with a median value of 1.65 (IQR 0.67–2.94) (*p* = 0.045). Current cannabis use was also associated with a greater proportion of participants with an adequate pain response phenotype (current use: 82.8% vs. past use: 28.6%, *p* = 0.004). The complete characteristics of participants’ cannabis use are presented in the Supplementary Table S3.

### Phenotypes

An adequate pain response phenotype was observed in 74 of the 100 participants; 25 had a positive screening test for possible CUD and 6 had at least one psychotic adverse event. The characteristics of participants according to each phenotype were investigated (Supplementary Table S4).

The data to establish pain response phenotype was missing for 6 participants who were consequently excluded from these analyses. Adequate pain response was not associated with any demographic characteristics, health status, comorbidities with the presence of neuropathic pain. Notably, there were no differences in concurrent pharmacotherapy between pain response groups. Current use was noted in 72 (97.3%) participants with a positive response phenotype and in 15 (75.0%) of non-responders (*p =* 0.004). Among participants with a defined pain response phenotype who completed the online survey (*n =* 43), an adequate pain response phenotype using the main questionnaire had a sensitivity of 89.1% (95% CI 74.6% **–** 97.0%) and specificity of 33.3% (4.3% **–** 77.7%) for adequate pain response based on the NRS values before and after cannabis use.

Some differences were noted among participants according to CUD screening test result. A lower prevalence of cardiovascular comorbidities was noted in participants with a positive CUD screening (20% vs. 45%, *p =* 0.025) as well as a lower prevalence of metabolic comorbidities (8.0% vs. 32%, *p =* 0.018) and lower body mass index (BMI) (25.1 vs. 28.7 kg/m^2^, *p =* 0.010). The only difference present regarding concurrent pharmacotherapy was lower benzodiazepine use in participants with positive screening for CUD (0 vs. 20.0%, *p =* 0.019).

Participants with a positive screening test for CUD were younger, had first used cannabis at a younger age and had lower pain duration. Lower benzodiazepine use, a decreased prevalence of cardiovascular and metabolic comorbidities as well as a BMI were also noted in participants with positive screening result.

Psychotic adverse events were not associated with any differences in demographic characteristics, or concurrent pharmacotherapy in the study participants. Metabolic comorbidities were more common among participants with psychotic adverse events. Notably, hallucinations were the only psychotic adverse event reported by the participants.

### Genetic association study

Saliva or blood sample was obtained for 77 participants. Statistically significant differences were observed between participants for whom DNA samples were obtained compared to participants without DNA samples (Supplementary Table S5). Participants with DNA samples were older than participants without DNA samples (50.4 vs. 40.1 years old, *p* < 0.001) and had longer chronic pain duration (median duration in years (IQR): 15.0 (7.9–23.6) vs. 8.5(4.2–18.0), *p =* 0.022). Patients with and without available DNA showed similar rates of pain response, psychotic adverse events, and CUDIT‑R scores.

Genotype validity assessment led to the exclusions of 10 variants from 9 different genes (*ABCB1*, *AKT1*, *CHRM3*, *CHRNA2*, *CNR2*, *CYP2C9*, *CYP3A5*, *FAAH*, *P2RX7*). The 18 remaining variants from 11 different genes (*BDNF*, *CNR1*, *CNR2*, *COMT*, *CYP2C9*, *FAAH*, *GABRA2*, *HES7*, *KAT2B*, *NRG1* and *OPMR1*) were at HWE following Holm-Bonferroni correction. HWE p-values, genotyping call rate of all variants (including those with call rate < 95%) and alternative allele frequency of the biallelic markers are presented in the Supplementary Table S6.

The three studied phenotypes according to the participants’ genotype for the different variants investigated are presented in Table 2. None of the variants investigated were associated with pain response phenotype or with CUD screening result. Two variants in the *CNR1* gene were associated with a statistically significant difference in the proportions of psychotic adverse events (before adjustment for multiple SNPs testing). Regarding the *CNR1* rs1049353 C > T variant, each additional T allele increased by sixfold the odds of having psychotic adverse events (odds ratio [OR] 6.1, 95% CI 1.7–27.9). Each additional C allele of the *CNR1* rs2023239 T > C intronic variant increased by threefold the odds of having psychotic adverse events ([OR] 3.5, 95% CI 1.5–9.4). These findings were not significant after adjustment for multiple SNPs testing.

**Table 2:** Response phenotype according to participants’ genotype

	**Pain response**				**CUDIT-R**				**Psychotic adverse events**			
	**Non-responder** (*N =* 16)	**Responder** (*N =* 57)	***p*-value**^*1*^	**Adjusted *p*-value**	**Negative** **(< 13)** (*N =* 60)	**Positive** **(≥ 13)** (*N =* 17)	***p*-value**^*2*^	**Adjusted *p*-value**	**Absence** (*N =* 71)	**Presence** (*N =* 6)	***p*-value**^*3*^	**Adjusted *p*-value**
Demographics												
Female sex, *n* (%)	12 (75.0)	39 (68.4)	0.84		46 (76.7)	9 (52.9)	0.072		50 (70.4)	5 (83.3)	0.67	
Median age in years (IQR)	55.29 (43.85, 64.29)	49.90 (38.10, 58.72)	0.21		51.2 (44.1, 62.5)	48.3 (34.1, 50.9)	**0.007**		50.4 (42.1, 59.9)	51.5 (40.1, 53.9)	0.79	
SNPs, n (%)												
*BDNF* (rs6265)			0.82	> 0.99			0.82	> 0.99			0.46	> 0.99
CC	10 (62)	37 (65)			37 (61.7)	12 (70.6)			44 (62.0)	5 (83.3)		
CT	6 (38)	19 (33)			22 (36.7)	5 (29.4)			26 (36.6)	1 (16.7)		
TT	0 (0)	1 (1.8)			1 (1.7)	0 (0.0)			1 (1.4)	0 (0.0)		
*CNR1* (rs806374)			0.79	> 0.99			> 0.99	> 0.99			0.38	> 0.99
TT	4 (25)	18 (32)			18 (30.0)	5 (29.4)			20 (28.2)	3 (50.0)		
TC	9 (56)	32 (56)			34 (56.7)	10 (58.8)			42 (59.2)	2 (33.3)		
CC	3 (19)	7 (12)			8 (13.3)	2 (11.8)			9 (12.7)	1 (16.7)		
*CNR1* (rs2023239)			0.15	> 0.99			> 0.99	> 0.99			**0.033**	0.49
TT	13 (81)	38 (67)			41 (68.3)	12 (70.6)			51 (71.8)	2 (33.3)		
TC	2 (12)	18 (32)			17 (28.3)	5 (29.4)			19 (26.8)	3 (50.0)		
CC	1 (6.2)	1 (1.8)			2 (3.3)	0 (0.0)			1 (1.4)	1 (16.7)		
*CNR1* (rs1049353)			0.45	> 0.99			0.92	> 0.99			**0.009**	0.14
CC	10 (62)	30 (53)			31 (51.7)	10 (58.8)			40 (56.3)	1 (16.7)		
CT	6 (38)	20 (35)			23 (38.3)	6 (35.3)			27 (38.0)	2 (33.3)		
TT	0 (0)	7 (12)			6 (10.0)	1 (5.9)			4 (5.6)	3 (50.0)		
*CNR1* (rs6454674)			0.81	> 0.99			0.74	> 0.99			0.78	> 0.99
TT	9 (56)	30 (53)			31 (51.7)	10 (58.8)			37 (52.1)	4 (66.7)		
TG	7 (44)	23 (40)			25 (41.7)	7 (41.2)			30 (42.3)	2 (33.3)		
GG	0 (0)	4 (7.0)			4 (6.7)	0 (0.0)			4 (5.6)	0 (0.0)		
*CNR1* (rs806368)			0.26	> 0.99			0.7	> 0.99			0.41	> 0.99
TT	4 (25)	26 (46)			26 (43.3)	6 (35.3)			29 (40.8)	3 (50.0)		
TC	11 (69)	26 (46)			30 (50.0)	9 (52.9)			37 (52.1)	2 (33.3)		
CC	1 (6.2)	5 (8.8)			4 (6.7)	2 (11.8)			5 (7.0)	1 (16.7)		
*CNR1* (rs806378)			0.68	> 0.99			0.52	> 0.99			> 0.99	> 0.99
CC	10 (62)	30 (53)			31 (51.7)	11 (64.7)			38 (53.5)	4 (66.7)		
CT	6 (38)	22 (39)			24 (40.0)	6 (35.3)			28 (39.4)	2 (33.3)		
TT	0 (0)	5 (8.8)			5 (8.3)	0 (0.0)			5 (7.0)	0 (0.0)		
*CNR1* (rs806380)			0.42	> 0.99			0.26	> 0.99			0.82	> 0.99
AA	9 (56)	25 (44)			25 (41.7)	11 (64.7)			32 (45.1)	4 (66.7)		
AG	7 (44)	25 (44)			29 (48.3)	5 (29.4)			32 (45.1)	2 (33.3)		
GG	0 (0)	7 (12)			6 (10.0)	1 (5.9)			7 (9.9)	0 (0.0)		
*CNR2* (rs2229579)^4^			> 0.99	> 0.99			0.082	> 0.99			> 0.99	> 0.99
GG	13 (81)	43 (80)			49 (86.0)	11 (64.7)			55 (80.9)	5 (83.3)		
GA	3 (19)	10 (19)			7 (12.3)	6 (35.3)			12 (17.6)	1 (16.7)		
AA	0 (0)	1 (1.9)			1 (1.8)	0 (0.0)			1 (1.5)	0 (0.0)		
*COMT* (rs4680)			0.53	> 0.99			0.73	> 0.99			0.87	> 0.99
GG	2 (12)	13 (23)			16 (26.7)	3 (17.6)			17 (23.9)	2 (33.3)		
GA	10 (62)	26 (46)			27 (45.0)	9 (52.9)			33 (46.5)	3 (50.0)		
AA	4 (25)	18 (32)			17 (28.3)	5 (29.4)			21 (29.6)	1 (16.7)		
*CYP2C9* (rs1799853)			0.78	> 0.99			0.36	> 0.99			0.17	> 0.99
CC	14 (88)	44 (77)			47 (78.3)	13 (76.5)			57 (80.3)	3 (50.0)		
CT	2 (12)	12 (21)			13 (21.7)	3 (17.6)			13 (18.3)	3 (50.0)		
TT	0 (0)	1 (1.8)			0 (0.0)	1 (5.9)			1 (1.4)	0 (0.0)		
*FAAH* (rs324420)			0.63	> 0.99			0.81	> 0.99			0.68	> 0.99
CC	11 (69)	43 (75)			44 (73.3)	12 (70.6)			52 (73.2)	4 (66.7)		
CA	5 (31)	13 (23)			15 (25.0)	5 (29.4)			18 (25.4)	2 (33.3)		
AA	0 (0)	1 (1.8)			1 (1.7)	0 (0.0)			1 (1.4)	0 (0.0)		
*GABRA2* (rs279858)^5^			0.46	> 0.99			0.2	> 0.99			0.82	> 0.99
TT	1 (6.2)	11 (20)			11 (18.6)	2 (11.8)			13 (18.6)	0 (0.0)		
TC	11 (69)	35 (62)			35 (59.3)	14 (82.4)			44 (62.9)	5 (83.3)		
CC	4 (25)	10 (18)			13 (22.0)	1 (5.9)			13 (18.6)	1 (16.7)		
*HES7* (rs1442849)			0.76	> 0.99			0.5	> 0.99			0.06	0.9
CC	7 (44)	26 (46)			25 (41.7)	10 (58.8)			32 (45.1)	3 (50.0)		
CT	7 (44)	27 (47)			30 (50.0)	6 (35.3)			35 (49.3)	1 (16.7)		
TT	2 (12)	4 (7.0)			5 (8.3)	1 (5.9)			4 (5.6)	2 (33.3)		
*KAT2B* (rs9829896)			0.16	> 0.99			0.4	> 0.99			> 0.99	> 0.99
CC	4 (25)	6 (11)			9 (15.0)	1 (5.9)			9 (12.7)	1 (16.7)		
CA	9 (56)	28 (49)			32 (53.3)	8 (47.1)			37 (52.1)	3 (50.0)		
AA	3 (19)	23 (40)			19 (31.7)	8 (47.1)			25 (35.2)	2 (33.3)		
*NRG1* (rs17664708)			> 0.99	> 0.99			0.78	> 0.99			0.61	> 0.99
CC	13 (81)	46 (81)			48 (80.0)	15 (88.2)			57 (80.3)	6 (100.0)		
CT	3 (19)	10 (18)			11 (18.3)	2 (11.8)			13 (18.3)	0 (0.0)		
TT	0 (0)	1 (1.8)			1 (1.7)	0 (0.0)			1 (1.4)	0 (0.0)		
*OPRM1* (rs510769)			0.29	> 0.99			0.35	> 0.99			0.76	> 0.99
CC	11 (69)	30 (53)			31 (51.7)	12 (70.6)			40 (56.3)	3 (50.0)		
CT	4 (25)	25 (44)			26 (43.3)	5 (29.4)			28 (39.4)	3 (50.0)		
TT	1 (6.2)	2 (3.5)			3 (5.0)	0 (0.0)			3 (4.2)	0 (0.0)		
*OPRM1* (rs1799971)			0.89	> 0.99			0.45	> 0.99			0.52	> 0.99
AA	10 (62)	38 (67)			40 (66.7)	10 (58.8)			47 (66.2)	3 (50.0)		
AG	5 (31)	17 (30)			17 (28.3)	7 (41.2)			21 (29.6)	3 (50.0)		
GG	1 (6.2)	2 (3.5)			3 (5.0)	0 (0.0)			3 (4.2)	0 (0.0)		

Statistically significant *p*-values are bolded

Adjusted *p*-values for SNPs and phenotype testing were calculated using Bonferroni correction for adjusting multilocus analyses with an effective number of 15 variants

*Abbreviations*: *Alt* alternative allele, *ref* reference allele

^1^Pearson’s Chi-squared test; Two Sample t-test

^2^Fisher’s exact test; Wilcoxon rank sum test

^3^Fisher’s exact test; Two Sample t-test

^4^Data available *N =* 74

^5^Data available *N =* 76

## Discussion

This retrospective genetic association study in patients with chronic pain who used cannabis or cannabinoids, describes the relationship between previously reported genetic variants and three main response phenotypes. More precisely, we assessed the relationship between different genetic variants and cannabis response in terms of pain response, CUD and psychotic adverse events. Our findings suggest that two variants of the *CNR1* gene (rs1049353 and rs2023239) could be associated with an increased rate of psychotic adverse events although these associations were not significant after adjustment for multiple SNPs testing. None of the studied variants were associated with CUD or pain response.

Previous studies have highlighted the significant inter-individual variability associated with THC use, both in terms of physiologic effects and pharmacokinetics parameters (Hunault et al. 2008; Liyanage et al. 2023). This variability, which applies to adverse events but also to pain response, underscores the importance of identifying genetic markers to personalize cannabis treatment. In 2022, an open-label non-randomized observational study by Poli et al*.* recruited 600 participants who received different cannabis preparations and reported for the first time variants associated with pain response (Poli et al. 2022). One of these variants, *ABCB1* rs1045642, was included as a candidate gene for this study but was unfortunately discarded due to insufficient call rate. The other two variants, *TRPV* rs8065080 and *UGT2B7* rs7438135, although both were genes of potential interests due to their role in the pharmacodynamics and pharmacokinetics of cannabis, were not included in our study due to lack of clinical studies investigating their impact on cannabis use and the studied phenotypes. However, Poli et al*.* identified the *CNR1* rs1049353 variant as a treatment discontinuation risk factor.

Previous literature highlights the role of genes implicated in the dopaminergic system (e.g., *COMT*) and psychosis induced by cannabis (Carvalho and Vieira-Coelho 2022), and offers insight into the mechanisms underlying an hypothetical link between *CNR1* alternative allele carrying and psychotic adverse events. *CNR1* encodes one of the two main cannabinoid receptors, cannabinoid receptor 1 (CB_1_), that is part of the G protein-coupled receptors (GPCRs) family of membrane proteins. CB_1_ is ubiquitous in the central nervous system and is distributed at a greater concentration in regions playing a key role in reward, cognition and emotions, such as the limbic areas, hippocampus and amygdala (Bloomfield et al. 2019). THC exhibits partial agonist activity of CB_1_ and is thought to be at the origin of most of the cannabis observed psychotropic effects (Shahbazi et al. 2020). Notably, THC could be responsible for the transient positive psychotic symptoms (e.g., hallucinations) that can result from cannabis use even in the absence of an underlying psychiatric disorder (Bloomfield et al. 2019). Data from animal studies suggests exogenous cannabinoids such as THC facilitate dopamine release from dopaminergic neurons via mechanisms involving CB_1_ (Bloomfield et al. 2016). While the data in humans is unclear, increased expression of CB_1_ on peripheral immune cells was documented in patients with multiple episodes of psychosis compared to healthy controls (Minichino et al. 2019).

*CNR1* rs1049353 polymorphism in exon 4 produces a synonymous variant in codon 453 (Thr453Thr). However, this synonymous SNP may impact mRNA stability and, consequently, affect CB_1_ receptor expression. Alteration in *CNR1* mRNA stability could therefore affect dopamine release in key dopaminergic regions associated with cannabis-induced psychosis. Moreover, an association of *CNR1* rs1049353 with psychotic adverse events could be an indirect association via linkage disequilibrium, as multiple *CNR1* haplotype blocks were documented in rs1049353 region (Hillard and Liu 2014). Similarly, evidence also suggests variable expression of CB_1_ receptor in presence of the *CNR1* rs2023239 polymorphism, also an intronic variant (Hutchison et al. 2008). Greater CB_1_ receptor density in peripheral lymphocytes for carriers of the alternative C allele was described in long-term daily cannabis users, like most of the participants in this study (Ketcherside et al. 2017). Interestingly, results from a pilot study using data from a placebo-controlled clinical trial investigating the impact of cannabis on driving performance, suggested that the *CNR1* rs1049353 and rs2023239 variants could increase subjective effects of acute cannabis intoxication (Murphy et al. 2021).

Surprisingly, despite most of the previous associations in the literature being with CUD (i.e., *CNR1* (rs806380, rs806378, rs806374, rs806368, rs2023239, rs1049353 and rs6454674) (Agrawal et al. 2009; Hindocha et al. 2020; Ashenhurst et al. 2017; Zuo et al. 2007; Palmer et al. 2019; Hartman et al. 2009), *FAAH* (rs324420) (Hindocha et al. 2020; Sipe et al. 2002; Tyndale et al. 2007), *GABRA2* (rs279858) (Agrawal et al. 2006), *HES7* (rs1442849) (Saffroy et al. 2019), *KAT2B* (rs9829896) (Johnson et al. 2016), *NRG1* (rs17664708) (Han et al. 2012) and *OPRM1* (rs1799971) (Schwantes-An et al. 2016)), none of the 18 variants included were associated with CUD in our study. An explanation for this discrepancy could be the studied population and the method employed to identify possible CUD among participants. In contrast to the previous studies that were conducted in adolescent or adult populations with non-medical use of cannabis, individuals included in this study used cannabis as means of self-management or as prescribed through health professionals. Limited evidence in the literature points towards altered test characteristics of the CUDIT-R in individuals with cannabis use for medical purposes (Loflin et al. 2018; Sagar et al. 2021). Similarly, Myers et al. recently reported that the CUDIT-R had worse performance among individuals who possessed a cannabis card compared to non-card holders (Myers et al. 2023). Higher frequency of use among medical users, like the majority of this study’s participants, could also have contributed to the decreased specificity of the CUDIT-R scale as many of its items are dependent on the frequency or intensity of use (Loflin et al. 2018).

While *TRPV1* and *UGT2B7* were not included at the time of the study, their inclusion alongside *ABCB1* as a multigene signal of analgesia would have been interesting as suggested by the results of the previously mentioned review by Kalak et al*.* (2026)*.* Two of the genes excluded from the analysis after genotype validity assessment (*AKT1* and *CYP2C9*) were the strongest markers of cannabis response according to their review.

This study has several limitations, primarily stemming from its retrospective design and the relatively small sample size, thereby limiting the findings to hypothesis‑generating interpretations. Despite the selection of candidate genes with previous positive association or based on our current understanding of the pharmacokinetics or pharmacodynamics of cannabinoids, the multiple testing involved in this study comes with the important risk of type I error. The retrospective nature of the study is obviously prone to recall bias. Furthermore, the small number of participants without active cannabis use implies significant selection bias and could have contributed to the low prevalence of both psychotic adverse events and negative pain response phenotypes observed since both of those could be motives to forgo cannabis use. The small sample size of this study, combined with the modest effect size of some of the previously reported variants, could also have contributed to our study being insufficiently powered to detect these associations.

## Conclusion

In summary, this retrospective genetic association study in patients with chronic pain raises the possibility that rs1049353 and rs2023239 may contribute to an increased rate of psychotic adverse events related to cannabis use in patients with chronic pain. This study did not replicate numerous previous findings as none of the variants studied were associated with possible CUD. The adequacy of the available screening tools for CUD in subpopulations of cannabis users remains uncertain and deserves greater attention considering the growing access to cannabis in chronic pain treatment. With those factors in mind, the results of this paper should be considered hypotheses. Finally, the role of these two *CNR1* variants should be further studied in an independent prospective cohort.

## Supplementary Information

Supplementary Material 1.

## Data Availability

Upon request and according to local access policies, the minimal dataset that would be necessary to interpret or replicate findings of the current study may be available from the corresponding author.

## Abbreviations

*ABCB1*: ATP Binding Cassette Subfamily B Member 1
*AKT1*: AKT Serine/Threonine Kinase 1
*BDNF*: Brain Derived Neurotrophic Factor
BMI: Body mass index
BPI: Brief Pain Inventory
CBD: Cannabidiol
*CHRM3*: Cholinergic Receptor Muscarinic 3
*CHRNA2*: Cholinergic Receptor Nicotinic Alpha 2 Subunit
*CNR1*: Cannabinoid receptor 1
*CNR2*: Cannabinoid receptor 2
*COMT*: Catechol-O-Methyltransferase
CUD: Cannabis use disorder
CUDIT-R: Cannabis Use Disorder Identification Test – Revised
*CYP2C9*: Cytochrome P450 Family 2 Subfamily C Member 9
*CYP3A5*: Cytochrome P450 Family 3 Subfamily A Member 5
DN4: Douleur Neuropathique 4
*FAAH*: Cytochrome P450 Family 3 Subfamily A Member 5
*GABRA2*: Gamma-Aminobutyric Acid Type A Receptor Subunit Alpha2
*HES7*: Hes Family BHLH Transcription Factor 7
HWE: Hardy-Weinberg Equilibrium
IQR: Interquartile range
*KAT2B*: Lysine Acetyltransferase 2B
*NRG1*: Neuregulin 1
NRS: Numerical pain rating scale
*OPRM1*: Opioid Receptor Mu 1
*P2RX7*: Purinergic Receptor P2X 7
SNP: Single-nucleotide polymorphism
THC: Delta9-tetrahydrocannabinol

## References

[CR1] Agrawal A, Edenberg HJ, Foroud T, Bierut LJ, Dunne G, Hinrichs AL, et al. Association of GABRA2 with drug dependence in the collaborative study of the genetics of alcoholism sample. Behav Genet. 2006;36(5):640–50. 16622805 10.1007/s10519-006-9069-4

[CR2] Agrawal A, Wetherill L, Dick DM, Xuei X, Hinrichs A, Hesselbrock V, et al. Evidence for association between polymorphisms in the Cannabinoid Receptor 1 (CNR1) gene and cannabis dependence. Am J Med Genet Part B Neuropsychiatr Genet off Publ Int Soc Psychiatr Genet. 2009;150B(5):736–40. 10.1002/ajmg.b.30881 PMC2703788 19016476

[CR3] Allan GM, Ramji J, Perry D, Ton J, Beahm NP, Crisp N, et al. Simplified guideline for prescribing medical cannabinoids in primary care. Can Fam Physician Med Fam Can. 2018;64(2):111–20. PMC5964385 29449241

[CR4] Allan GM, Finley CR, Ton J, Perry D, Ramji J, Crawford K, et al. Systematic review of systematic reviews for medical cannabinoids. Can Fam Physician. 2018;64(2):e78-94. 29449262 PMC5964405

[CR5] Ashenhurst JR, Harden KP, Mallard TT, Corbin WR, Fromme K. Developmentally specific associations between CNR1 genotype and Cannabis use across emerging adulthood. J Stud Alcohol Drugs. 2017;78(5):686–95. 28930056 10.15288/jsad.2017.78.686 PMC5675419

[CR6] Babayeva M, Loewy ZG. Cannabis pharmacogenomics: a path to personalized medicine. Curr Issues Mol Biol. 2023;45(4):3479–514. 37185752 10.3390/cimb45040228 PMC10137111

[CR7] Bloomfield MAP, Ashok AH, Volkow ND, Howes OD. The effects of Δ9-tetrahydrocannabinol on the dopamine system. Nature. 2016;539(7629):369–77. 27853201 10.1038/nature20153 PMC5123717

[CR8] Bloomfield MAP, Hindocha C, Green SF, Wall MB, Lees R, Petrilli K, et al. The neuropsychopharmacology of cannabis: a review of human imaging studies. Pharmacol Ther. 2019;195:132–61. 30347211 10.1016/j.pharmthera.2018.10.006 PMC6416743

[CR9] Bonn-Miller MO, Heinz AJ, Smith EV, Bruno R, Adamson S. Preliminary development of a brief Cannabis Use disorder screening tool: the Cannabis use disorder identification test short-form. Cannabis Cannabinoid Res. 2016;1(1):252–61. 28861497 10.1089/can.2016.0022 PMC5531365

[CR10] Bouhassira D, Attal N, Alchaar H, Boureau F, Brochet B, Bruxelle J, et al. Comparison of pain syndromes associated with nervous or somatic lesions and development of a new neuropathic pain diagnostic questionnaire (DN4). Pain. 2005;114(1–2):29–36. 15733628 10.1016/j.pain.2004.12.010

[CR11] Breivik H, Borchgrevink PC, Allen SM, Rosseland LA, Romundstad L, Hals EKB, et al. Assessment of pain. Br J Anaesth. 2008;101(1):17–24. 18487245 10.1093/bja/aen103

[CR12] Carvalho C, Vieira-Coelho MA. Cannabis induced psychosis: a systematic review on the role of genetic polymorphisms. Pharmacol Res. 2022;181:106258. 35588917 10.1016/j.phrs.2022.106258

[CR13] Cleeland CS, Ryan KM. Pain assessment: global use of the brief pain inventory. Ann Acad Med Singap. 1994;23(2):129–38. 8080219

[CR14] Cohen SP, Vase L, Hooten WM. Chronic pain: an update on burden, best practices, and new advances. Lancet Lond Engl. 2021;397(10289):2082–97. 10.1016/S0140-6736(21)00393-7 34062143

[CR15] DNAgentotek. The prepIT^TM^•L2P reagent laboratory protocol for manual purification of DNA. Ottawa, Canada. Available from: https://www.dnagenotek.com/globaldocs/ifu/prepit-lp2/PD-HB-2.pdf.

[CR16] Ferreira-Valente MA, Pais-Ribeiro JL, Jensen MP. Validity of four pain intensity rating scales. Pain. 2011;152(10):2399–404. 21856077 10.1016/j.pain.2011.07.005

[CR17] Foll BL, Tang VM, Rueda S, Trick LV, Boileau I. Cannabis use disorder: from neurobiology to treatment. J Clin Invest. 2024;134(20). Available from: https://www.jci.org/articles/view/172887. Cited 14 Jan 2026. 10.1172/JCI172887 PMC11473150 39403927

[CR18] GBD 2016 Disease and Injury Incidence and Prevalence Collaborators. 2016 Global, regional, and national incidence, prevalence, and years lived with disability for 328 diseases and injuries for 195 countries, 1990–2016: a systematic analysis for the Global Burden of Disease Study 2016. Lancet Lond Engl. 2017;390(10100):1211–59. 10.1016/S0140-6736(17)32154-2 PMC5605509 28919117

[CR19] Godbout-Parent M, Nguena Nguefack HL, Angarita-Fonseca A, Audet C, Bernier A, Zahlan G, et al. Prevalence of cannabis use for pain management in Quebec: a post-legalization estimate among generations living with chronic pain. Can J Pain. 2022;6(1):65–77. 35694144 10.1080/24740527.2022.2051112 PMC9176231

[CR20] Government of Canada PS and PC. Chronic pain in Canada : laying a foundation for action.: H134–5/2019E-PDF - Government of Canada Publications - Canada.ca. 2002. Available from: https://publications.gc.ca/site/eng/9.875857/publication.html. Cited 20 Jun 2025.

[CR21] Han S, Yang BZ, Kranzler HR, Oslin D, Anton R, Farrer LA, et al. Linkage analysis followed by association show NRG1 associated with cannabis dependence in African-Americans. Biol Psychiatry. 2012;72(8):637–44. 22520967 10.1016/j.biopsych.2012.02.038 PMC3699339

[CR22] Harris PA, Taylor R, Thielke R, Payne J, Gonzalez N, Conde JG. Research electronic data capture (REDCap)--a metadata-driven methodology and workflow process for providing translational research informatics support. J Biomed Inform. 2009;42(2):377–81. 18929686 10.1016/j.jbi.2008.08.010 PMC2700030

[CR23] Harris PA, Taylor R, Minor BL, Elliott V, Fernandez M, O’Neal L, et al. The REDCap consortium: building an international community of software platform partners. J Biomed Inform. 2019;95:103208. 31078660 10.1016/j.jbi.2019.103208 PMC7254481

[CR24] Hartman CA, Hopfer CJ, Haberstick B, Rhee SH, Crowley TJ, Corley RP, et al. The association between Cannabinoid receptor 1 gene (CNR1) and Cannabis dependence symptoms in adolescents and young adults. Drug Alcohol Depend. 2009;104(1–2):11–6. 19443135 10.1016/j.drugalcdep.2009.01.022 PMC2769509

[CR25] Herdman M, Gudex C, Lloyd A, Janssen MF, Kind P, Parkin D, et al. Development and preliminary testing of the new five-level version of EQ-5D (EQ-5D-5L). Qual Life Res. 2011;20(10):1727–36. 21479777 10.1007/s11136-011-9903-x PMC3220807

[CR26] Hillard CJ, Liu QS. Endocannabinoid signaling in the etiology and treatment of major depressive illness. Curr Pharm Des. 2014;20(23):3795–811. 24180398 10.2174/13816128113196660735 PMC4002665

[CR27] Hindocha C, Freeman TP, Schafer G, Gardner C, Bloomfield MAP, Bramon E, et al. Acute effects of cannabinoids on addiction endophenotypes are moderated by genes encoding the CB1 receptor and FAAH enzyme. Addict Biol. 2020;25(3):e12762. 31013550 10.1111/adb.12762

[CR28] Holland PM, Abramson RD, Watson R, Gelfand DH. Detection of specific polymerase chain reaction product by utilizing the 5’––3’ exonuclease activity of Thermus aquaticus DNA polymerase. Proc Natl Acad Sci U S A. 1991;88(16):7276–80. 1871133 10.1073/pnas.88.16.7276 PMC52277

[CR29] Hryhorowicz S, Walczak M, Zakerska-Banaszak O, Słomski R, Skrzypczak-Zielińska M. Pharmacogenetics of cannabinoids. Eur J Drug Metab Pharmacokinet. 2018;43(1):1–12. 28534260 10.1007/s13318-017-0416-z PMC5794848

[CR30] Hunault CC, Mensinga TT, de Vries I, Kelholt-Dijkman HH, Hoek J, Kruidenier M, et al. Delta-9-tetrahydrocannabinol (THC) serum concentrations and pharmacological effects in males after smoking a combination of tobacco and cannabis containing up to 69 mg THC. Psychopharmacology Berl. 2008;201(2):171–81. 18695931 10.1007/s00213-008-1260-2

[CR31] Hutchison KE, Haughey H, Niculescu M, Schacht J, Kaiser A, Stitzel J, et al. The incentive salience of alcohol: translating the effects of genetic variant in CNR1. Arch Gen Psychiatry. 2008;65(7):841–50. 18606956 10.1001/archpsyc.65.7.841 PMC2856651

[CR32] Johnson EO, Hancock DB, Levy JL, Gaddis NC, Page GP, Glasheen C, et al. KAT2B polymorphism identified for drug abuse in African Americans with regulatory links to drug abuse pathways in human prefrontal cortex. Addict Biol. 2016;21(6):1217–32. 26202629 10.1111/adb.12286 PMC4724343

[CR33] Kalak M, Brylak-Błaszków A, Błaszków Ł, Kalak T, Kalak M, Brylak-Błaszków A, et al. Medical Marijuana and Treatment Personalization: The Role of Genetics and Epigenetics in Response to THC and CBD. Genes. 2025;16(12). Available from: https://www.mdpi.com/2073-4425/16/12/1487. Cited 13 Jan 2026. 10.3390/genes16121487 PMC12732823 41465160

[CR34] Ketcherside A, Noble LJ, McIntyre CK, Filbey FM. Cannabinoid receptor 1 gene by Cannabis use interaction on CB1 receptor density. Cannabis Cannabinoid Res. 2017;2(1):202–9. 29082317 10.1089/can.2017.0007 PMC5628563

[CR35] Li J, Ji L. Adjusting multiple testing in multilocus analyses using the eigenvalues of a correlation matrix. Heredity. 2005;95(3):221–7. 16077740 10.1038/sj.hdy.6800717

[CR36] Liyanage M, Nikanjam M, Capparelli EV, Suhandynata RT, Fitzgerald RL, Marcotte TD, et al. Variable delta-9-tetrahydrocannabinol pharmacokinetics and pharmacodynamics after Cannabis smoking in regular users. Ther Drug Monit. 2023;45(5):689–96. 37199428 10.1097/FTD.0000000000001104

[CR37] Loflin M, Babson K, Browne K, Bonn-Miller M. Assessment of the validity of the CUDIT-R in a subpopulation of cannabis users. Am J Drug Alcohol Abuse. 2018;44(1):19–23. 29058471 10.1080/00952990.2017.1376677

[CR38] Minichino A, Senior M, Brondino N, Zhang SH, Godwlewska BR, Burnet PWJ, et al. Measuring disturbance of the endocannabinoid system in psychosis. JAMA Psychiatry. 2019;76(9):914–23. 31166595 10.1001/jamapsychiatry.2019.0970 PMC6552109

[CR39] Murphy T, Matheson J, Mann RE, Brands B, Wickens CM, Tiwari AK, et al. Influence of Cannabinoid receptor 1 genetic variants on the subjective effects of smoked Cannabis. Int J Mol Sci. 2021;22(14):7388. 34299009 10.3390/ijms22147388 PMC8307475

[CR40] Myers MG, Ganoczy D, Walters HM, Pfeiffer PN, Ilgen MA, Bohnert KM. Assessing the diagnostic utility of the Cannabis Use Disorder Identification Test - Revised (CUDIT-R) among veterans with medical and non-medical cannabis use. Drug Alcohol Depend. 2023;1(247):109876. 10.1016/j.drugalcdep.2023.109876 37130467

[CR41] Palmer RHC, McGeary JE, Knopik VS, Bidwell LC, Metrik JM. CNR1 and FAAH variation and affective states induced by marijuana smoking. Am J Drug Alcohol Abuse. 2019;45(5):514–26. 31184938 10.1080/00952990.2019.1614596 PMC6931041

[CR42] Poli P, Peruzzi L, Maurizi P, Mencucci A, Scocca A, Carnevale S, et al. The pharmacogenetics of cannabis in the treatment of chronic pain. Genes. 2022;13(10):1832. 36292717 10.3390/genes13101832 PMC9601332

[CR43] QIAGEN. Protocol: DNA purification from whole blood or bone marrow using the puregene blood kit. Germany. Available from: https://www.qiagen.com/us/resources/resourcedetail?id=a9e6a609-4600-4b03-afbd-974318590ce5&lang=en.

[CR44] R Core Team. A language and environment for statistical computing. 2023. Available from: https://www.r-project.org/. Cited 20 Jun 2025.

[CR45] Saffroy R, Lafaye G, Desterke C, Ortiz-Tudela E, Amirouche A, Innominato P, et al. Several clock genes polymorphisms are meaningful risk factors in the development and severity of cannabis addiction. Chronobiol Int. 2019;36(1):122–34. 30526093 10.1080/07420528.2018.1523797

[CR46] Sagar KA, Dahlgren MK, Smith RT, Lambros AM, Gruber SA. Assessing cannabis use disorder in medical cannabis patients: interim analyses from an observational, longitudinal study. Cannabis. 2021;4(2):47–59. 37287530 10.26828/cannabis/2021.02.004 PMC10212242

[CR47] Santé Canada. Un plan d’action pour la douleur au Canada. 2021. Available from: chrome-extension://efaidnbmnnnibpcajpcglclefindmkaj/https://www.canada.ca/content/dam/hc-sc/documents/corporate/about-health-canada/public-engagement/external-advisory-bodies/canadian-pain-task-force/report-2021-rapport/report-rapport-2021-fra.pdf.

[CR48] Schoeler T, Baldwin JR, Martin E, Barkhuizen W, Pingault JB. Assessing rates and predictors of cannabis-associated psychotic symptoms across observational, experimental and medical research. Nature Mental Health. 2024;2(7):865–76. 39005547 10.1038/s44220-024-00261-x PMC11236708

[CR49] Schulz P, Walser A, Meyer JJ, Kubli A, Garrone G. French translation of the Stanford Sleepiness Scale and use of this scale of sedation following a single dose of midazolam or amitriptyline. Agressol Rev Int Physio-Biol Pharmacol Appl Aux Eff Agression. 1983;24(8):357–9. 6660359

[CR50] Schwantes-An TH, Zhang J, Chen LS, Hartz SM, Culverhouse RC, Chen X, et al. Association of the OPRM1 variant rs1799971 (A118G) with non-specific liability to substance dependence in a collaborative de novo meta-analysis of European-ancestry cohorts. Behav Genet. 2016;46(2):151–69. 26392368 10.1007/s10519-015-9737-3 PMC4752855

[CR51] Shahbazi F, Grandi V, Banerjee A, Trant JF. Cannabinoids and cannabinoid receptors: the story so far. iScience. 2020;23(7):101301. 32629422 10.1016/j.isci.2020.101301 PMC7339067

[CR52] Sipe JC, Chiang K, Gerber AL, Beutler E, Cravatt BF. A missense mutation in human fatty acid amide hydrolase associated with problem drug use. Proc Natl Acad Sci U S A. 2002;99(12):8394–9. 12060782 10.1073/pnas.082235799 PMC123078

[CR53] Tyndale RF, Payne JI, Gerber AL, Sipe JC. The fatty acid amide hydrolase C385A (P129T) missense variant in cannabis users: studies of drug use and dependence in Caucasians. Am J Med Genet Part B Neuropsychiatr Genet off Publ Int Soc Psychiatr Genet. 2007;144B(5):660–6. 10.1002/ajmg.b.30491 17290447

[CR54] Visibelli A, Peruzzi L, Poli P, Scocca A, Carnevale S, Spiga O, et al. Supporting machine learning model in the treatment of chronic pain. Biomedicines. 2023;11(7). Available from: https://www.mdpi.com/2227-9059/11/7/1776. Cited 14 Jan 2026. 10.3390/biomedicines11071776 PMC10376077 37509416

[CR55] Visibelli A, Finetti R, Roncaglia B, Poli P, Spiga O, Santucci A. Predicting therapy dropout in chronic pain management: a machine learning approach to cannabis treatment. Front Artif Intell. 2025;8. Available from: https://www.frontiersin.org/journals/artificial-intelligence/articles/ 10.3389/frai.2025.1557894/full. Cited 14 Jan 2026. 10.3389/frai.2025.1557894 PMC11882547 40051572

[CR56] Volkow ND, Baler RD, Compton WM, Weiss SRB. Adverse health effects of marijuana use. N Engl J Med. 2014;370(23):2219–27. 24897085 10.1056/NEJMra1402309 PMC4827335

[CR57] Wang L, Hong PJ, May C, Rehman Y, Oparin Y, Hong CJ, et al. Medical cannabis or cannabinoids for chronic non-cancer and cancer related pain: a systematic review and meta-analysis of randomised clinical trials. BMJ. 2021;374:n1034. 34497047 10.1136/bmj.n1034

[CR58] Whirl-Carrillo M, McDonagh EM, Hebert JM, Gong L, Sangkuhl K, Thorn CF, et al. Pharmacogenomics knowledge for personalized medicine. Clin Pharmacol Ther. 2012;92(4):414–7. 22992668 10.1038/clpt.2012.96 PMC3660037

[CR59] Xie F, Pullenayegum E, Gaebel K, Bansback N, Bryan S, Ohinmaa A, et al. A time trade-off-derived value set of the EQ-5D-5L for Canada. Med Care. 2016;54(1):98–105. 26492214 10.1097/MLR.0000000000000447 PMC4674140

[CR60] Zuo L, Kranzler HR, Luo X, Covault J, Gelernter J. CNR1 variation modulates risk for drug and alcohol dependence. Biol Psychiatry. 2007;62(6):616–26. 17509535 10.1016/j.biopsych.2006.12.004

