# Supplementary material for: Pharmacogenetic association study of cannabis use in chronic pain
Source: J Cannabis Res. 2026 Feb 14;8:41. doi: 10.1186/s42238-026-00408-w (PMC13011771; doi:10.1186/s42238-026-00408-w)
Supplement: Supplementary file 1 — Supplementary Material 1. [file 42238_2026_408_MOESM1_ESM.docx]

Supplementary Table S1. Genetic variants associated with response phenotypes to cannabis or potentially linked to its pharmacokinetics and pharmacodynamic

| Gene symbol | Gene name | Gene ID | Locus^¥^ | SNP^¥^ | Alleles^¥^ | MAF^¥^ | Start position (pb)^¥^ | Variant type | Population | Biogeographical group | Phenotype | Ref |
| --- | --- | --- | --- | --- | --- | --- | --- | --- | --- | --- | --- | --- |
| ABCB1 | *ATP binding cassette subfamily B member 1* | 5243 | 7q21.12 | rs1045642 | A>G,T | 0.49570 | 87509329 | Synonymous | Patients with cannabis dependence vs. controls | European | CUD | (1) |
| AKT1 | *AKT serine/threonine kinase 1* | 207 | 14q32.33 | rs2494732 | T>C,G | 0.49865 | 104772855 | Intron | Patients with first-episode psychosis vs. controls | Mixed | Psychotic effects (via risk of psychotic disorders in cannabis users) | (2) |
| BDNF | *Brain derived neurotrophic factor* | 627 | 11p14.1 | rs6265 | C>T | 0.19488 | 27658369 | Missense | Participants with psychotic experiences or psychotic disorders vs. controls | Mixted | Psychotic effects (via the influence the risk of psychotic experiences and is also involved in dopaminergic systems) | (3) |
| CHRM3 | *Cholinergic receptor muscarinic 3* | 1131 | 1q43 | rs74722579 | G>C | 0.06143 | 239401207 | Intron | Long-term cannabis users | Mixted | Psychotic effects (via association with cannabis-induced hallucinations) | (4)§ |
|  |  |  |  | rs115455482 | C>T | 0.03752 | 239399460 | Intron |  |  |  |  |
| CHRNA2 | *Cholinergic receptor nicotinic α2* | 1135 | 8p21.2 | rs56372821 | G>A | 0.12850 | 27436500 | Intron | Participants with CUD vs. controls | Mixed | CUD | (5)§ |
| CNR1 | *Cannabinoid receptor 1* | 1268 | 6q15 | rs806380 | A>G | 0.24882 | 88154934 | Intron | Participants with cannabis  Dependence (DSM-III-R) vs. controls | Mixed | CUD | (6) |
|  |  |  |  | rs806378 | C>T | 0.19652 | 88149832 | Intron | Cannabis users without schizophrenia/psychosis or substance abuse problems | Mixed | CUD (via salience to cannabis appetitive cues) | (7) |
|  |  |  |  | rs806374 | T>C | 0.38187 | 88147601 | Intron | University students | Mixed | CUD (via increased odds of use around age 18) | (8) |
|  |  |  |  | rs806368 | T>C | 0.20711 | 88140381 | 3’ UTR | Participants with substance dependence vs. controls. | Mixed | CUD (via increased risk of drug dependence) | (9) |
|  |  |  |  | rs2023239 | T>C | 0.20769 | 88150763 | Intron | Adult regular cannabis users | Mixed | Cannabis effects (via influence on mood states related to cannabis use) | (10) |
|  |  |  |  | rs1049353 | C>T | 0.21412 | 88143916 | Synonymous | Participants with ≥ 1 DSM-IV cannabis dependence symptom vs. controls without | Mixed | CUD (via increased risk of presenting cannabis dependence symptoms) | (11) |
|  |  |  |  | rs6454674 | T>G | 0.31613 | 88163211 | Intron | Participants with substance dependence vs. controls. | Mixed | CUD (via increased risk of drug dependence) | (9) |
| CNR2 | *Cannabinoid receptor 2* | 1269 | 1p36.11 | rs2501432 | T>C,G | 0.38339 | 23875430 | Missense | HEK293 cells (pharmacodynamic study) | N/A | Cannabis effects (via reduced function of CB2 polymorphic  receptors when activated by endogenous cannabinoids) | (12) |
|  |  |  |  | rs2229579 | G>A,T | 0.08163 | 23874672 | Missense |  |  |  |  |
| COMT | *Catechol-O-methyltransferase* | 1312 | 22q11.21 | rs4680 | G>A | 0.46102 | 19963748 | Missense | Patients  with a psychotic disorder vs. controls (13)  Prospective birth cohort (14) | Unknown (13)  Mixed (14) | Psychotic effects (cannabis-induced hallucinations) (13)  Psychotic effects (via increased psychotic symptoms and risk of schizophreniform disorder in cannabis users (14) | (13, 14) |
| CYP 2C9 | *cytochrome P450 family 2 subfamily C member 9* | 1559 | 10q23.33 | rs1799853 | C>T | 0.09202 | 94942290 | Missense | Healthy volunteers | European | Cannabis effects (via influence on pharmacokinetics of orally administered Δ9-tetrahydrocannabinol (THC)) | (15) |
|  |  |  |  | rs1057910 | A>C,G | 0.06807 | 94981296 | Missense |  |  |  |  |
| CYP 3A5 | *cytochrome P450 family 3 subfamily A member 5* | 1577 | 7q22.1 | rs776746 | T>C | 0.11835 | 99672916 | Splice Acceptor |  |  |  |  |
| FAAH | *Fatty acid amide hydrolase* | 2166 | 1p33 | *rs324420 | C>A | 0.23609 | 46405089 | Missense | Cannabis users without schizophrenia/psychosis or substance abuse problems (7)  Patients at a medical screening clinic (16)  Healthy volunteers (17) | Mixed (7)  Mixed (16)  American (17) | CUD (bias toward appetitive stimuli to cannabis) (7)  CUD (via increased street drug use and problem drug/alcohol  use) (16)  CUD (via increased of cannabis dependence (DSM IV) (17) | (7, 16, 17) |
|  |  |  |  | rs2295633 | A>G,T | 0.40425 | 46408711 | Intron | Patients with first-episode psychosis vs. controls | Mixed | Psychotic effects (via greater risk of presenting psychosis in subjects with cannabis use) | (18) |
| GABRA2 | *Gamma-aminobutyric acid type A receptor alpha2 subunit* | 2555 | 4p12 | rs279858 | T>C | 0.40385 | 46312576 | Synonymous | Individuals from families of alcohol dependent probands | Mixed | CUD (via association with with marijuana  Dependence (DSM-III-R) | (19) |
| HES7 | *Hes family bHLH transcription factor 7* | 84667 | 17p13.1 | rs1442849 | C>T | 0.32513 | 8120803 | 3’ UTR | Patients presenting for excessive cannabis consumption and cannabis use disorder (DSM-IV) vs. controls | European | CUD (via overrepresentation in cannabis users, earlier cannabis use and larger weekly consumption) | (20) |
| KAT2B | *Lysine acetyltransferase 2B* | 8850 | 3p24.3 | *rs9829896 | C>A,T | 0.25332 | 20135980 | Intron | Participants with drug abuse (opioids, cocaine, marijuana, stimulants, and/or other drugs) vs. controls | Mixed | CUD (via influence on risk of drug abuse in african americans) | (21)§ |
| NRG1 | *Neuregulin 1* | 3084 | 8p12 | rs17664708 | C>T | 0.09482 | 32579499 | Intron | Participants initialy recruited for genetic studies of cocaine and  opioid dependence | Mixed | CUD (via assessement by Semi-Structured Assessment for Drug  Dependence and Alcoholism (SSADDA)) | (22)§ |
| OPRM1 | *Opioid receptor mu 1* | 4988 | 6q25.2 | *rs1799971 | A>G | 0.18842 | 154039662 | Missense | Participants with DSM-IV defined  alcohol, cannabis, cocaine, or opioid dependence. | European | CUD (via modulation of substance dependence liability on multiple substances, including cannabis, as assesseded by the DSM-IV) | (23) |
|  |  |  |  | rs510769 | C>T | 0.25050 | 154040884 | Intron | Regular cannabis users | Unknown | Cannabis effects (via impact on subjective cannabis effects and THC blood levels) | (24) |
| P2RX7 | *purinergic receptor P2X 7* | 5027 | 12q24.31 | rs7958311 | G>A,C | 0.25591 | 121167552 | Missense | Participants without a psychiatric disorder | Mixed | Psychotic effects (via influence on regular cannabis use and risk of psychotic experiences in a dose response fashion) | (25)§ |

Abbreviations: MAF = Minor allele frequency; SNP = single nucleotide polymorphism; CUD = cannabis use disorder.

¥ Data from genome version GRCh38/hg38 (reference > alternative allele); in european ancestry population; <http://ncbi.nlm.nih.gov/snp/>

* Genetic variants reported in ClinPGx data base (<https://www.clinpgx.org/> ) (n = 4)

§ GWAS study

References:

1. Benyamina A, Bonhomme-Faivre L, Picard V, Sabbagh A, Richard D, Blecha L, et al. Association between ABCB1 C3435T polymorphism and increased risk of cannabis dependence. Prog Neuropsychopharmacol Biol Psychiatry. 2009;33(7):1270-4.

2. Di Forti M, Iyegbe C, Sallis H, Kolliakou A, Falcone MA, Paparelli A, et al. Confirmation that the AKT1 (rs2494732) genotype influences the risk of psychosis in cannabis users. Biol Psychiatry. 2012;72(10):811-6.

3. Kirli U, Binbay T, Drukker M, Elbi H, Kayahan B, Gokcelli DK, et al. Is BDNF-Val66Met polymorphism associated with psychotic experiences and psychotic disorder outcome? Evidence from a 6 years prospective population-based cohort study. Am J Med Genet B Neuropsychiatr Genet. 2019;180(2):113-21.

4. Cheng Z, Phokaew C, Chou YL, Lai D, Meyers JL, Agrawal A, et al. A regulatory variant of CHRM3 is associated with cannabis-induced hallucinations in European Americans. Transl Psychiatry. 2019;9(1):309.

5. Demontis D, Rajagopal VM, Thorgeirsson TE, Als TD, Grove J, Leppala K, et al. Genome-wide association study implicates CHRNA2 in cannabis use disorder. Nat Neurosci. 2019;22(7):1066-74.

6. Agrawal A, Wetherill L, Dick DM, Xuei X, Hinrichs A, Hesselbrock V, et al. Evidence for association between polymorphisms in the cannabinoid receptor 1 (CNR1) gene and cannabis dependence. Am J Med Genet B Neuropsychiatr Genet. 2009;150B(5):736-40.

7. Hindocha C, Freeman TP, Schafer G, Gardner C, Bloomfield MAP, Bramon E, et al. Acute effects of cannabinoids on addiction endophenotypes are moderated by genes encoding the CB1 receptor and FAAH enzyme. Addict Biol. 2020;25(3):e12762.

8. Ashenhurst JR, Harden KP, Mallard TT, Corbin WR, Fromme K. Developmentally Specific Associations Between CNR1 Genotype and Cannabis Use Across Emerging Adulthood. J Stud Alcohol Drugs. 2017;78(5):686-95.

9. Zuo L, Kranzler HR, Luo X, Covault J, Gelernter J. CNR1 variation modulates risk for drug and alcohol dependence. Biol Psychiatry. 2007;62(6):616-26.

10. Palmer RHC, McGeary JE, Knopik VS, Bidwell LC, Metrik JM. CNR1 and FAAH variation and affective states induced by marijuana smoking. Am J Drug Alcohol Abuse. 2019;45(5):514-26.

11. Hartman CA, Hopfer CJ, Haberstick B, Rhee SH, Crowley TJ, Corley RP, et al. The association between cannabinoid receptor 1 gene (CNR1) and cannabis dependence symptoms in adolescents and young adults. Drug Alcohol Depend. 2009;104(1-2):11-6.

12. Carrasquer A, Nebane NM, Williams WM, Song ZH. Functional consequences of nonsynonymous single nucleotide polymorphisms in the CB2 cannabinoid receptor. Pharmacogenet Genomics. 2010;20(3):157-66.

13. Henquet C, Rosa A, Delespaul P, Papiol S, Fananas L, van Os J, et al. COMT ValMet moderation of cannabis-induced psychosis: a momentary assessment study of 'switching on' hallucinations in the flow of daily life. Acta Psychiatr Scand. 2009;119(2):156-60.

14. Caspi A, Moffitt TE, Cannon M, McClay J, Murray R, Harrington H, et al. Moderation of the effect of adolescent-onset cannabis use on adult psychosis by a functional polymorphism in the catechol-O-methyltransferase gene: longitudinal evidence of a gene X environment interaction. Biol Psychiatry. 2005;57(10):1117-27.

15. Sachse-Seeboth C, Pfeil J, Sehrt D, Meineke I, Tzvetkov M, Bruns E, et al. Interindividual variation in the pharmacokinetics of Delta9-tetrahydrocannabinol as related to genetic polymorphisms in CYP2C9. Clin Pharmacol Ther. 2009;85(3):273-6.

16. Sipe JC, Chiang K, Gerber AL, Beutler E, Cravatt BF. A missense mutation in human fatty acid amide hydrolase associated with problem drug use. Proc Natl Acad Sci U S A. 2002;99(12):8394-9.

17. Tyndale RF, Payne JI, Gerber AL, Sipe JC. The fatty acid amide hydrolase C385A (P129T) missense variant in cannabis users: studies of drug use and dependence in Caucasians. Am J Med Genet B Neuropsychiatr Genet. 2007;144B(5):660-6.

18. Bioque M, Mas S, Costanzo MC, Cabrera B, Lobo A, Gonzalez-Pinto A, et al. Gene-environment interaction between an endocannabinoid system genetic polymorphism and cannabis use in first episode of psychosis. Eur Neuropsychopharmacol. 2019;29(6):786-94.

19. Agrawal A, Edenberg HJ, Foroud T, Bierut LJ, Dunne G, Hinrichs AL, et al. Association of GABRA2 with drug dependence in the collaborative study of the genetics of alcoholism sample. Behav Genet. 2006;36(5):640-50.

20. Saffroy R, Lafaye G, Desterke C, Ortiz-Tudela E, Amirouche A, Innominato P, et al. Several clock genes polymorphisms are meaningful risk factors in the development and severity of cannabis addiction. Chronobiol Int. 2019;36(1):122-34.

21. Johnson EO, Hancock DB, Levy JL, Gaddis NC, Page GP, Glasheen C, et al. KAT2B polymorphism identified for drug abuse in African Americans with regulatory links to drug abuse pathways in human prefrontal cortex. Addict Biol. 2016;21(6):1217-32.

22. Han S, Yang BZ, Kranzler HR, Oslin D, Anton R, Farrer LA, et al. Linkage analysis followed by association show NRG1 associated with cannabis dependence in African Americans. Biol Psychiatry. 2012;72(8):637-44.

23. Schwantes-An TH, Zhang J, Chen LS, Hartz SM, Culverhouse RC, Chen X, et al. Association of the OPRM1 Variant rs1799971 (A118G) with Non-Specific Liability to Substance Dependence in a Collaborative de novo Meta-Analysis of European-Ancestry Cohorts. Behav Genet. 2016;46(2):151-69.

24. Bourgault Z, Matheson J, Mann RE, Brands B, Wickens CM, Tiwari AK, et al. Mu opioid receptor gene variant modulates subjective response to smoked cannabis. Am J Transl Res. 2022;14(1):623-32.

25. Boks MP, He Y, Schubart CD, Gastel WV, Elkrief L, Huguet G, et al. Cannabinoids and psychotic symptoms: A potential role for a genetic variant in the P2X purinoceptor 7 (P2RX7) gene. Brain Behav Immun. 2020;88:573-81.

Supplementary Table S2. Probe and primer designs for genotyping with TaqMan

| Gene | Variant | Probe type | Probe design | Primer Type | Primer Design |
| --- | --- | --- | --- | --- | --- |
| ABCB1 | rs1045642 | REF | TGCCCT+CA+C+A+AT+CTCTTCCT | Forward | CATTAGGCAGTGACTCGATGAA |
|  |  | A to G | TGCCCTCA+C+G+AT+CTCTTCCT | Reverse | TGAGAACATTGCCTATGGAGAC |
|  |  | A to T | TGCCCT+CA+C+T+AT+CTCTTCCT | - | - |
| AKT1 | rs2494732 | REF | ATCCCA+GC+T+A+CAGGCTACAC | Forward | TATCAGTGTAGTCTGGGAGGTG |
|  |  | T to C | ATCCCAGC+T+G+CAGGCTACAC | Reverse | CTTTCAGGGCTGCTCAAGAA |
|  |  | T to G | ATCCCAGC+T+C+CAGGCTACAC | - | - |
| BDNF | rs6265 | REF | CTCTTCT+ATC+A+C+G+TGT+TCGAAAGT | Forward | TTCATTGGGCCGAACTTTCT |
|  |  | C to T | CTCTT+CT+ATC+A+T+G+TGT+TCGAAAGT | Reverse | CCCAAGGCAGGTTCAAGAG |
| CHRM3 | rs74722579 | REF | ACCCAGGTA+C+C+AG+TTTCTT | Forward | TAGCAGTTATCACGTGTTAGT |
|  |  | G to C | ACCCAGGTA+C+G+AG+TTTCTT | Reverse | TGAAGAGTAGAATGGGAGTTTC |
|  | rs115455482 | REF | CTCAAACTTAT+TC+AT+C+C+T+AAATGA | Forward | TGTTAAGCTACGGATGAAATACTGA |
|  |  | C to T | CTCAAACTTAT+TC+AT+C+T+T+AAATGA | Reverse | TGATGAAGCTGGAGAATGGC |
| CHRNA2 | rs56372821 | REF | TGCCT+C+G+GCCTCCAAAG | Forward | GGATGGTCTCGATCTCCTGA |
|  |  | G to A | TGC+CT+C+A+GCCTCCAAAG | Reverse | GAGATGATTTAAAGTATATGGGAGTGCC |
| CNR1 | rs806380 | REF | CAAA+GTT+TT+CA+A+TTAA+GTAAAAGC | Forward | CGCCCAGAAAGCAATTA |
|  |  | A to G | CAAA+GTT+TT+CA+G+TTAA+GTAAAAGC | Reverse | GACCAAATCTCATATGCAAGA |
|  | rs806378 | REF | CCTCATC+ACG+T+C+G+TATAATCAGGA | Forward | CCCAGCACATCCCTCTATTAC |
|  |  | C to T | CCTCATC+ACG+T+T+G+TA+TAATCAGGA | Reverse | GATGCCTCTGTGTTTGGAGA |
|  | rs806374 | REF | ACAGGCATA+AA+GT+G+T+TA+AATGTGG | Forward | GACAGGACACAGAGTCAATGAA |
|  |  | T to C | ACAGGCATA+AA+GT+G+C+TA+AATGTGG | Reverse | CAGTGATGAGATCCTGGAAGTG |
|  | rs806368 | REF | AGAAACTC+TC+CC+A+T+CC+GAAA | Forward | CCAGTAGGCCTAGTACAGTCAA |
|  |  | T to C | AACTCTC+CC+A+C+CCGAAA | Reverse | GTTTGAGCAGTGGCCTACA |
|  | rs2023239 | REF | CAGGA+CC+A+T+GT+AA+GGAACAG | Forward | AAGTATGGGTGGGAGTTGAAAG |
|  |  | T to C | CAGGA+CC+A+C+GT+AAGGAACAG | Reverse | GGACACAGAAGACAGTCACAATA |
|  | rs1049353 | REF | CAATCTT+GA+C+C+GT+GCTCTTGA | Forward | GCAGACGTGTCTGTGGAC |
|  |  | C to T | TGGCAATCTTGA+C+T+GT+GCTCTTGA | Reverse | GCAAACAATGCAGCCAGTG |
|  | rs6454674 | REF | AAGCAA+TA+AC+T+T+TC+ACCCAGAATT | Forward | ACATAGATCAATTCAGACTTCTCCA |
|  |  | T to G | AAGCAATAAC+T+G+TC+ACCCAGAATT | Reverse | TGAAAGATTTGGTTATAGCTTCTTGA |
| CNR2 | rs2229579 | REF | CTTCTTCCAG+T+G+AGCCAGG | Forward | AGCCAAGCTGCCAATGAA |
|  |  | G to A | CTTCTTCC+AG+T+A+AG+CCAGG | Reverse | TGCTGTGTTGTGCACTCTT |
|  |  | G to T | CTTCTTCC+AG+T+T+AG+CCAGG | - | - |
|  | rs2501432 | REF | TGTCCTCCCAC+C+A+ACTCCG | Forward | TTTGCCTCTGACCCAAGG |
|  |  | T to C | TGTCCTCCCAC+C+GACTCC | Reverse | TATGCTCTACGGAGTGGAGAG |
|  |  | T to G | TGTCCTCCCAC+C+CACTCC | - | - |
| COMT | rs4680 | REF | TCCTTC+A+C+GCCAGCGAA | Forward | GAGGCTCATCACCATCGAG |
|  |  | G to A | TCCTTC+A+T+GCCAGCGAA | Reverse | TTTCCAGGTCTGACAACGG |
| CYP2C9 | rs1799853 | REF | TGAACA+C+G+GTCCTCAATGCT | Forward | TCAGCAATGGAAAGAAATGGAAG |
|  |  | C to T | TGAACA+C+A+GT+CCTCAATGCT | Reverse | TTTCTCAACTCCTCCACAAGG |
|  | rs1057910 | REF | TCCAGAG+ATA+C+A+TT+GACCTTCTCC | Forward | CCACATGCCCTACACAGATG |
|  |  | A to C | TCCAGAGATA+C+C+TT+GACCTTCTC | Reverse | TTAATGTCACAGGTCACTGCAT |
|  |  | A to G | TCCAGAGATA+C+G+TT+GACCTTCTC | - | - |
| CYP3A5 | rs776746 | REF | TTGTCTT+TC+A+A+TA+TC+TCTTCCCTG | Forward | CCACCCAAGGCTTCATA |
|  |  | T to C | TTGTCTTTC+A+G+TA+TCTCTTCCCTG | Reverse | GAATGCTCTACTGTCATTTCTAAC |
| FAAH | rs324420 | REF | TCTCAGGCC+CCA+AGGCAG | Forward | CAACTGTGTGACCTCCTATCTG |
|  |  | C to A | TCTCAGGC+C+ACA+AGGCAG | Reverse | GAGGCAGAGCATACCTTGTAG |
|  | rs2295633 | REF | TCCCAC+AGG+G+T+CACAGTT | Forward | TCCATCCCTGGCATCCT |
|  |  | A to G | TCCCACAGGG+C+CACAGTT | Reverse | CAGGGCTAAAGTTCTCCATCAA |
|  |  | A to T | TCCCAC+AGG+G+A+CACAGTT | - | - |
| GABRA2 | rs279858 | REF | AGCTACT+GA+TT+T+T+TT+CCCATTGTG | Forward | AGCAGAGTCCCATCATCCT |
|  |  | T to C | AGCTACTGA+TT+T+C+TT+CCCATTGTG | Reverse | GGTCCTATGAATATCCTTCGACTAAA |
| HES7 | rs1442849 | REF | TGGCTTGG+C+C+TCTGAGAT | Forward | TCTCTCTCACCTGGCTAACA |
|  |  | C to T | TGGCTT+GG+C+T+TC+TGAGAT | Reverse | TACGGGTACTAGAGTGGGAATG |
| KAT2B | rs9829896 | REF | CCTACGTTG+G+C+GGTGTTCTA | Forward | GAATTGGTCTATCTCACCACCA |
|  |  | C to A | CCTACGT+TG+G+A+GGTGTTCTA | Reverse | AGGATCTTTGGAAGTCATTCTATCT |
|  |  | C to T | CCTACGT+TG+G+T+GGTGTTCTA | - | - |
| NRG1 | rs17664708 | REF | TCATGGTTTT+C+C+ACCTCCATTC | Forward | CAGCCTCTGGGATTGAACTT |
|  |  | C to T | TCATGGTT+TT+C+T+AC+CTCCATTC | Reverse | TCACCTCCATACCCTCCTATTC |
| OPRM1 | rs510769 | REF | TGTATATT+CA+AAT+A+C+TA+CATGTGA | Forward | CAATTGCACTGATGCCTTGG |
|  |  | C to T | TGTATATT+CA+AAT+A+T+TA+CATGTGA | Reverse | CGTGATCATGGAGGGACTG |
|  | rs1799971 | REF | TCGGACAGG+T+T+GC+CATCTAAG | Forward | GTGTGGAAGCTCTCAGTA |
|  |  | A to G | TCGGACAGG+T+C+GCCATCTAAG | Reverse | AAACAGATATATGGCATTTCACAT |
| P2RX7 | rs7958311 | REF | CATCACTGC+C+G+TC+CCAAATAC | Forward | TACTGGGACTGCAACCTAGA |
|  |  | G to A | CATCACT+GC+C+A+TC+CC+AAATAC | Reverse | TAGCCAGGGTACAAGGACA |
|  |  | G to C | CATCACTGC+C+C+TC+CCAAATAC | - | - |

ABCB1 = ATP binding cassette subfamily B member 1; AKT1 = AKT serine/threonine kinase 1; BDNF = Brain derived neurotrophic factor; CHRM3 = Cholinergic receptor muscarinic 3; CHRNA2 = Cholinergic receptor nicotinic α2; CNR1 = Cannabinoid receptor 1; CNR2 = Cannabinoid receptor 2; COMT = Catechol-O-methyltransferase; CYP 2C9 = cytochrome P450 family 2 subfamily C member 9; CYP 3A5 = cytochrome P450 family 3 subfamily A member 5; FAAH = Fatty acid amide hydrolase; GABRA2 = Gamma-aminobutyric acid type A receptor alpha2 subunit; HES7 = Hes family bHLH transcription factor 7; KAT2B = Lysine acetyltransferase 2B; NRG1 = Neuregulin 1; OPRM1 = Opioid receptor mu 1; P2RX7 = purinergic receptor P2X 7

Supplementary Table S3. Cannabis use characteristics

|  | Overall (N = 100) | Current use (N = 92) | Past use (N = 8) |
| --- | --- | --- | --- |
| Main method of use, n (%) |  |  |  |
| Inhaled | 44 (44%) | 43 (47%) | 1 (12%) |
| Oral | 45 (45%) | 40 (43%) | 5 (62%) |
| Other or more than one | 11 (11%) | 9 (9.8%) | 2 (25%) |
| Frequency of use, n (%) |  |  |  |
| ≤ Weekly | 4 (4.0%) | 3 (3.3%) | 1 (12%) |
| More than once per week | 15 (15%) | 13 (14%) | 2 (25%) |
| Daily | 27 (27%) | 26 (28%) | 1 (12%) |
| More than once daily | 54 (54%) | 50 (54%) | 4 (50%) |
| Mean duration of cannabis use in years (SD) | 5.11 (6.89) | 5.51 (7.05) | 0.47 (0.52) |
| Mean age at first cannabis use in years (SD) | 23.73 (15.39) | 23.42 (14.97) | 27.25 (20.58) |
| Mean age at initiation of daily cannabis use in years (SD) | 34.27 (17.12) | 33.89 (17.11) | 39.77 (17.75) |
| Unknown | 8 | 6 | 2 |
| Mean quantity used by inhalation in grams per day of use (SD) | 1.22 (1.05) | 1.22 (1.05) | NA (NA) |
| Unknown | 67 | 59 | 8 |
| Mean THC content of cannabis used by inhalation in % (SD) | 13.12 (9.23) | 13.12 (9.23) | NA (NA) |
| Unknown | 62 | 54 | 8 |
| Mean CBD content of cannabis used by inhalation in % (SD) | 4.23 (5.79) | 4.23 (5.79) | NA (NA) |
| Unknown | 61 | 53 | 8 |
| THC/CBD content (inhaled), n (%) |  |  |  |
| THC > CBD*^1^* | 27 (71%) | 27 (71%) | 0 (NA%) |
| CBD > THC*^2^* | 4 (11%) | 4 (11%) | 0 (NA%) |
| THC ≈ CBD | 7 (18%) | 7 (18%) | 0 (NA%) |
| Unknown | 62 | 54 | 8 |
| THC/CBD content (oral), n (%) |  |  |  |
| THC > CBD*^1^* | 5 (10%) | 5 (11%) | 0 (0%) |
| CBD > THC*^2^* | 34 (69%) | 31 (70%) | 3 (60%) |
| THC ≈ CBD | 10 (20%) | 8 (18%) | 2 (40%) |
| Unknown | 51 | 48 | 3 |
| Median amount of THC ingested per day of use in mg (IQR) | 13.66 (39.98) | 14.34 (41.83) | 6.65 (6.36) |
| Unknown | 55 | 51 | 4 |
| Median amount of CBD ingested per day of use in mg (IQR) | 21.55 (12.40, 48.15) | 22.00 (12.30, 48.20) | 13.00 (12.49, 40.00) |
| Unknown | 56 | 53 | 3 |
| *^1^* Containing at least twice the amount of THC compared to CBD; *^2^* Containing at least twice the amount of CBD compared to THC.  Abbreviations: THC = Δ9-tetrahydrocannabinol; CBD = cannabidiol; | | | |

Supplementary Table S4. Participant characteristics and studied phenotypes

|  | Pain response | | | | CUDIT-R | | | Psychotic adverse events | | |
| --- | --- | --- | --- | --- | --- | --- | --- | --- | --- | --- |
|  | Non-responder (N = 20) | Responder (N = 74) | | p-value*^1^* | Negative (< 13) (N = 75) | Positive (≥ 13) (N = 25) | p-value*^1^* | Absence (N = 94) | Presence (N = 6) | p-value*^2^* |
| Demographics | | | | | | | | | | |
| Female sex, n (%) | 15 (75.0%) | 48 (64.9%) | | 0.39 | 53 (70.7%) | 14 (56.0%) | 0.18 | 62 (66.0%) | 5 (83.3%) | 0.66 |
| Age (years) | 50.95 (13.12) | 46.94 (13.15) | | 0.23 | 51.01 (12.07) | 39.06 (11.97) | <0.001 | 47.95 (13.19) | 49.06 (11.96) | 0.84 |
| Ethnic origin*^3^,* n (%) |  |  | |  |  |  |  |  |  |  |
| European | 20 (100.0%) | 70 (94.6%) | | >0.99 | 71 (95.9%) | 24 (96.0%) | 0.69 | 89 (95.7%) | 6 (100.0%) | >0.99 |
| Latino | 0 (0.0%) | 2 (2.7%) | |  | 2 (2.7%) | 0 (0.0%) |  | 2 (2.2%) | 0 (0.0%) |  |
| Other | 0 (0.0%) | 2 (2.7%) | |  | 1 (1.4%) | 1 (4.0%) |  | 2 (2.2%) | 0 (0.0%) |  |
| Chronic pain and health status | | | | | | | | | | |
| DN4 score ≥ 4, n (%) | 16 (80.0%) | 48 (64.9%) | | 0.20 | 50 (66.7%) | 15 (60.0%) | 0.55 | 61 (64.9%) | 4 (66.7%) | >0.99 |
| Pain duration (years)*^4^*, n (%) | 20.0 (7.4, 24.4) | 11.8 (6.9, 21.2) | | 0.27 | 15.0 (9.0, 23.7) | 7.5 (4.4, 13.2) | 0.004 | 12.0 (6.5, 22.0) | 17.3 (10.9, 20.0) | 0.58 |
| Median BPI pain severity (IQR)*^3^* | 5.62 (4.00, 6.31) | 5.00 (3.50, 6.00) | | 0.21 | 5.25 (3.50, 6.25) | 5.25 (3.25, 6.00) | 0.63 | 5.25 (3.50, 6.25) | 4.00 (4.00, 4.25) | 0.18 |
| Median BPI pain interference (IQR)*^3^* | 3.38 (1.66, 6.19) | 3.88 (2.00, 5.50) | | 0.86 | 3.74 (1.66, 5.08) | 4.12 (2.00, 6.00) | 0.32 | 3.74 (1.59, 5.50) | 5.38 (3.88, 5.57) | 0.17 |
| EQ-5D-5L Mobility |  |  | 0.26 |  | |  | 0.58 |  |  | 0.50 |
| No problems | 3 (15.0%) | 27 (36.5%) |  | 26 (34.7%) | | 6 (24.0%) |  | 30 (31.9%) | 2 (33.3%) |  |
| Slight problems | 6 (30.0%) | 17 (23.0%) |  | 16 (21.3%) | | 8 (32.0%) |  | 24 (25.5%) | 0 (0.0%) |  |
| Moderate problems | 9 (45.0%) | 25 (33.8%) |  | 28 (37.3%) | | 9 (36.0%) |  | 33 (35.1%) | 4 (66.7%) |  |
| Severe problems | 2 (10.0%) | 3 (4.1%) |  | 4 (5.3%) | | 1 (4.0%) |  | 5 (5.3%) | 0 (0.0%) |  |
| Unable to walk about | 0 (0.0%) | 2 (2.7%) |  | 1 (1.3%) | | 1 (4.0%) |  | 2 (2.1%) | 0 (0.0%) |  |
| EQ-5D-5L Self-care |  |  | 0.96 |  | |  | 0.91 |  |  | 0.63 |
| No problems | 12 (60.0%) | 45 (60.8%) |  | 46 (61.3%) | | 16 (64.0%) |  | 59 (62.8%) | 3 (50.0%) |  |
| Slight problems | 7 (35.0%) | 23 (31.1%) |  | 23 (30.7%) | | 7 (28.0%) |  | 27 (28.7%) | 3 (50.0%) |  |
| Moderate problems | 1 (5.0%) | 3 (4.1%) |  | 3 (4.0%) | | 2 (8.0%) |  | 5 (5.3%) | 0 (0.0%) |  |
| Severe problems | 0 (0.0%) | 1 (1.4%) |  | 1 (1.3%) | | 0 (0.0%) |  | 1 (1.1%) | 0 (0.0%) |  |
| Unable to wash or dress | 0 (0.0%) | 2 (2.7%) |  | 2 (2.7%) | | 0 (0.0%) |  | 2 (2.1%) | 0 (0.0%) |  |
| EQ-5D-5L Usual Activities*^3^* |  |  | 0.68 |  | |  | 0.24 |  |  | 0.36 |
| No problems | 3 (15.0%) | 19 (26.0%) |  | 15 (20.3%) | | 8 (32.0%) |  | 23 (24.7%) | 0 (0.0%) |  |
| Slight problems | 4 (20.0%) | 9 (12.3%) |  | 13 (17.6%) | | 1 (4.0%) |  | 14 (15.1%) | 0 (0.0%) |  |
| Moderate problems | 9 (45.0%) | 31 (42.5%) |  | 33 (44.6%) | | 9 (36.0%) |  | 37 (39.8%) | 5 (83.3%) |  |
| Severe problems | 4 (20.0%) | 11 (15.1%) |  | 11 (14.9%) | | 6 (24.0%) |  | 16 (17.2%) | 1 (16.7%) |  |
| Unable to do usual activities | 0 (0.0%) | 3 (4.1%) |  | 2 (2.7%) | | 1 (4.0%) |  | 3 (3.2%) | 0 (0.0%) |  |
| EQ-5D-5L Pain/Discomfort |  |  | 0.83 |  | |  | 0.79 |  |  | 0.26 |
| No pain/discomfort | 0 (0.0%) | 1 (1.4%) |  | 1 (1.3%) | | 0 (0.0%) |  | 1 (1.1%) | 0 (0.0%) |  |
| Slight pain/discomfort | 3 (15.0%) | 18 (24.3%) |  | 19 (25.3%) | | 4 (16.0%) |  | 22 (23.4%) | 1 (16.7%) |  |
| Moderate pain/discomfort | 10 (50.0%) | 33 (44.6%) |  | 33 (44.0%) | | 13 (52.0%) |  | 41 (43.6%) | 5 (83.3%) |  |
| Severe pain/discomfort | 7 (35.0%) | 21 (28.4%) |  | 21 (28.0%) | | 8 (32.0%) |  | 29 (30.9%) | 0 (0.0%) |  |
| Extreme pain/discomfort | 0 (0.0%) | 1 (1.4%) |  | 1 (1.3%) | | 0 (0.0%) |  | 1 (1.1%) | 0 (0.0%) |  |
| EQ-5D-5L Anxiety/Depression |  |  | 0.16 |  | |  | 0.004 |  |  | 0.40 |
| Not anxious/depressed | 11 (55.0%) | 22 (29.7%) |  | 33 (44.0%) | | 2 (8.0%) |  | 34 (36.2%) | 1 (16.7%) |  |
| Slightly anxious/depressed | 4 (20.0%) | 25 (33.8%) |  | 22 (29.3%) | | 9 (36.0%) |  | 29 (30.9%) | 2 (33.3%) |  |
| Moderately anxious/depressed | 2 (10.0%) | 19 (25.7%) |  | 13 (17.3%) | | 8 (32.0%) |  | 19 (20.2%) | 2 (33.3%) |  |
| Severely anxious/depressed | 2 (10.0%) | 4 (5.4%) |  | 5 (6.7%) | | 3 (12.0%) |  | 8 (8.5%) | 0 (0.0%) |  |
| Extremely anxious/depressed | 1 (5.0%) | 4 (5.4%) |  | 2 (2.7%) | | 3 (12.0%) |  | 4 (4.3%) | 1 (16.7%) |  |
| Median EQ-5D-5L index (IQR)*^3^* | 0.68 (0.47, 0.76) | 0.70 (0.51, 0.82) | | 0.54 | 0.71 (0.52, 0.82) | 0.54 (0.40, 0.78) | 0.11 | 0.68 (0.48, 0.82) | 0.70 (0.64, 0.76) | 0.89 |
| Mean EQ VAS (SD) | 57.8 (19.6) | 63.2 (19.6) | | 0.20 | 62.6 (21.1) | 59.5 (20.8) | 0.38 | 62.0 (21.3) | 58.3 (14.7) | 0.45 |
| Mean BMI in kg/m2 (SD)*^4^* | 28.6 (6.5) | 27.4 (6.2) | | 0.22 | 28.7 (6.2) | 25.1 (5.3) | 0.010 | 28.0 (6.1) | 24.5 (6.5) | 0.27 |
| Cannabis use characteristics | | | | | | | | | | |
| Main method of use, n (%) |  |  | | 0.22 |  |  | <0.001 |  |  | 0.84 |
| Inhaled | 6 (30.0%) | 36 (48.6%) | |  | 25 (33.3%) | 19 (76.0%) |  | 42 (44.7%) | 2 (33.3%) |  |
| Oral | 10 (50.0%) | 31 (41.9%) | |  | 40 (53.3%) | 5 (20.0%) |  | 42 (44.7%) | 3 (50.0%) |  |
| Other or more than one | 4 (20.0%) | 7 (9.5%) | |  | 10 (13.3%) | 1 (4.0%) |  | 10 (10.6%) | 1 (16.7%) |  |
| Frequency of use, n (%) |  |  | | >0.99 |  |  | 0.49 |  |  | 0.50 |
| ≤ Weekly | 1 (5.0%) | 3 (4.1%) | |  | 4 (5.3%) | 0 (0.0%) |  | 4 (4.3%) | 0 (0.0%) |  |
| More than once per week | 3 (15.0%) | 11 (14.9%) | |  | 13 (17.3%) | 2 (8.0%) |  | 14 (14.9%) | 1 (16.7%) |  |
| Daily | 5 (25.0%) | 20 (27.0%) | |  | 20 (26.7%) | 7 (28.0%) |  | 24 (25.5%) | 3 (50.0%) |  |
| More than once daily | 11 (55.0%) | 40 (54.1%) | |  | 38 (50.7%) | 16 (64.0%) |  | 52 (55.3%) | 2 (33.3%) |  |
| Mean age at first cannabis use in years (SD) | 26.20 (17.19) | 22.49 (14.74) | | 0.21 | 25.83 (16.63) | 17.42 (8.34) | 0.005 | 23.75 (15.20) | 23.50 (19.88) | 0.67 |
| Past medical history, n (%) | | | | | | | | | | |
| Musculoskeletal | 20 (100.0%) | 71 (95.9%) | | >0.99 | 73 (97.3%) | 24 (96.0%) | >0.99 | 91 (96.8%) | 6 (100.0%) | >0.99 |
| Psychiatric | 13 (65.0%) | 53 (71.6%) | | 0.57 | 50 (66.7%) | 20 (80.0%) | 0.21 | 65 (69.1%) | 5 (83.3%) | 0.67 |
| Gastrointestinal | 12 (60.0%) | 39 (52.7%) | | 0.56 | 42 (56.0%) | 11 (44.0%) | 0.30 | 49 (52.1%) | 4 (66.7%) | 0.68 |
| Neurologic | 7 (35.0%) | 32 (43.2%) | | 0.51 | 32 (42.7%) | 8 (32.0%) | 0.35 | 39 (41.5%) | 1 (16.7%) | 0.40 |
| Cardiovascular | 10 (50.0%) | 28 (37.8%) | | 0.33 | 34 (45.3%) | 5 (20.0%) | 0.025 | 38 (40.4%) | 1 (16.7%) | 0.40 |
| Respiratory | 8 (40.0%) | 26 (35.1%) | | 0.69 | 24 (32.0%) | 12 (48.0%) | 0.15 | 35 (37.2%) | 1 (16.7%) | 0.41 |
| Metabolic | 5 (25.0%) | 19 (25.7%) | | 0.95 | 24 (32.0%) | 2 (8.0%) | 0.018 | 21 (22.3%) | 5 (83.3%) | 0.004 |
| Cancer | 3 (15.0%) | 7 (9.5%) | | 0.44 | 7 (9.3%) | 3 (12.0%) | 0.71 | 9 (9.6%) | 1 (16.7%) | 0.48 |
| Concurrent pharmacotherapy, n (%) | | | | | | | | | | |
| Antidepressants | 13 (65.0%) | 39 (52.7%) | | 0.33 | 41 (54.7%) | 14 (56.0%) | 0.91 | 50 (53.2%) | 5 (83.3%) | 0.22 |
| Acetaminophen | 8 (40.0%) | 22 (29.7%) | | 0.38 | 27 (36.0%) | 6 (24.0%) | 0.27 | 32 (34.0%) | 1 (16.7%) | 0.66 |
| NSAIDs | 8 (40.0%) | 22 (29.7%) | | 0.38 | 24 (32.0%) | 8 (32.0%) | >0.99 | 30 (31.9%) | 2 (33.3%) | >0.99 |
| Opioids | 7 (35.0%) | 22 (29.7%) | | 0.65 | 27 (36.0%) | 5 (20.0%) | 0.14 | 30 (31.9%) | 2 (33.3%) | >0.99 |
| Antiepileptics | 7 (35.0%) | 18 (24.3%) | | 0.34 | 22 (29.3%) | 6 (24.0%) | 0.61 | 25 (26.6%) | 3 (50.0%) | 0.35 |
| Muscle relaxants | 3 (15.0%) | 14 (18.9%) | | >0.99 | 15 (20.0%) | 4 (16.0%) | 0.77 | 19 (20.2%) | 0 (0.0%) | 0.59 |
| Benzodiazepines | 4 (20.0%) | 9 (12.2%) | | 0.46 | 15 (20.0%) | 0 (0.0%) | 0.019 | 15 (16.0%) | 0 (0.0%) | 0.59 |
| Stimulants | 1 (5.0%) | 7 (9.5%) | | >0.99 | 5 (6.7%) | 4 (16.0%) | 0.22 | 9 (9.6%) | 0 (0.0%) | >0.99 |
| Z drugs/benzodiazepine like | 0 (0.0%) | 6 (8.1%) | | 0.34 | 4 (5.3%) | 3 (12.0%) | 0.36 | 5 (5.3%) | 2 (33.3%) | 0.055 |
| Biologics/DMARDs | 1 (5.0%) | 2 (2.7%) | | 0.52 | 2 (2.7%) | 1 (4.0%) | >0.99 | 3 (3.2%) | 0 (0.0%) | >0.99 |
| Pain management using cannabis only | 2 (10.0%) | 5 (6.8%) | 0.64 | 5 (6.7%) | | 3 (12.0%) | 0.41 | 8 (8.5%) | 0 (0.0%) | >0.99 |
| Phenotype validation, n (%) | | | | | | | | | | |
| Cannabis use |  |  | | 0.004 |  |  |  |  |  |  |
| Current use | 15 (75.0%) | 72 (97.3%) | |  |  |  |  |  |  |  |
| Past use | 5 (25.0%) | 2 (2.7%) | |  |  |  |  |  |  |  |
| Online survey pain response (≥ 30%) |  |  | | 0.19 |  |  |  |  |  |  |
| Non-responder | 2 (33.3%) | 4 (10.8%) | |  |  |  |  |  |  |  |
| Responder | 4 (66.7%) | 33 (89.2%) | |  |  |  |  |  |  |  |
| *^1^* Pearson's Chi-squared test; Two Sample t-test; Fisher's exact test *^2^* Fisher's exact test; Two Sample t-test *^3^* Data available N = 99; *^4^* Data available N = 98;  Abbreviations: DMARDs = Disease-modifying antirheumatic drugs; NSAIDs = Non-Steroidal Anti-Inflammatory Drugs; | | | | | | | | | | |

Supplementary Table S5. Participant characteristics and phenotypes according to availability of DNA

|  | DNA (N = 77) | No DNA (N = 23) | p-value*^1^* |
| --- | --- | --- | --- |
| Demographics | | | |
| Female sex, n (%) | 55 (71.4%) | 12 (52.2%) | 0.085 |
| Mean age in years (SD) | 50.4 (12.7) | 40.1 (11.1) | <0.001 |
| Ethnic origin, n (%)*^2^* |  |  |  |
| European | 74 (97.3%) | 21 (91.3%) | 0.23 |
| Latino | 1 (1.3%) | 1 (4.3%) |  |
| Other | 1 (1.3%) | 1 (4.3%) |  |
| Cannabis use, chronic pain and health status | | | |
| Cannabis use, n (%) |  |  | >0.99 |
| Current use | 71 (92.2%) | 21 (91.3%) |  |
| Past use | 6 (7.8%) | 2 (8.7%) |  |
| Mean pain duration in years (SD)*^3^* | 15.0 (7.9, 23.6) | 8.5 (4.2, 18.0) | 0.022 |
| Mean BPI pain severity (SD)*^2^* | 5.25 (3.50, 6.06) | 5.25 (2.50, 6.12) | 0.75 |
| Mean BPI pain interference (SD)*^4^* | 3.62 (1.56, 5.60) | 3.88 (2.62, 5.12) | 0.96 |
| Mean EQ-5D-5L index (SD)*^2^* | 0.70 (0.48, 0.81) | 0.66 (0.52, 0.83) | 0.62 |
| Mean EQ VAS (SD) | 62.6 (20.8) | 59.1 (21.8) | 0.62 |
| Phenotypes, n (%) | | | |
| Pain response (≥ 30%)*^5^* | 57 (78.1%) | 17 (81.0%) | >0.99 |
| Psychotic adverse events | 6 (7.8%) | 0 (0.0%) | 0.33 |
| CUDIT-R ≥ 13 | 17 (22.1%) | 8 (34.8%) | 0.22 |
| *^1^* Pearson's Chi-squared test; Wilcoxon rank sum test; Fisher's exact test *^2^* Data available N = 99; *^3^* Data available N = 98; *^4^* Data available N = 97; *^5^* Data available N = 94; | | | |

Supplementary Table S6. Hardy-Weinberg equilibrium tests

| Gene (variant) | p-value | adjusted p-value | Callrate (%) | Ref | Alt | Alt allele frequency | Kept in the analyses |
| --- | --- | --- | --- | --- | --- | --- | --- |
| ABCB1 (rs1045642) | < 0.001 | 0.001 | 64.9 | A | G/T | - | No |
| AKT1 (rs2494732) | 0.081 | 1.000 | 70.1 | T | C/G | - | No |
| BDNF (rs6265) | 0.283 | 1.000 | 100.0 | C | T | 0.188 | Yes |
| CHRM3 (rs115455482) | 0.002 | 0.035 | 100.0 | C | T | 0.442 | No |
| CHRM3 (rs74722579) | < 0.001 | < 0.001 | 98.7 | G | C | 0.118 | No |
| CHRNA2 (rs56372821) | 1.000 | 1.000 | 100.0 | G | A | 0.000 | No |
| CNR1 (rs1049353) | 0.575 | 1.000 | 100.0 | C | T | 0.279 | Yes |
| CNR1 (rs2023239) | 1.000 | 1.000 | 100.0 | T | C | 0.169 | Yes |
| CNR1 (rs6454674) | 0.766 | 1.000 | 100.0 | T | G | 0.260 | Yes |
| CNR1 (rs806368) | 0.304 | 1.000 | 100.0 | T | C | 0.331 | Yes |
| CNR1 (rs806374) | 0.162 | 1.000 | 100.0 | T | C | 0.416 | Yes |
| CNR1 (rs806378) | 1.000 | 1.000 | 100.0 | C | T | 0.260 | Yes |
| CNR1 (rs806380) | 1.000 | 1.000 | 100.0 | A | G | 0.312 | Yes |
| CNR2 (rs2229579) | 0.545 | 1.000 | 96.1 | G | A | 0.333 | Yes |
| CNR2 (rs2501432) | < 0.001 | 0.001 | 84.4 | T | C/G | - | No |
| COMT (rs4680) | 0.648 | 1.000 | 100.0 | G | A | 0.481 | Yes |
| CYP2C9 (rs1057910) | < 0.001 | < 0.001 | 29.9 | A | C | 0.348 | No |
| CYP2C9 (rs1799853) | 1.000 | 1.000 | 100.0 | C | T | 0.117 | Yes |
| CYP3A5 (rs776746) | 0.174 | 1.000 | 100.0 | T | C | 0.955 | No |
| FAAH (rs2295633) | < 0.001 | 0.001 | 54.5 | A | G/T | - | No |
| FAAH (rs324420) | 1.000 | 1.000 | 100.0 | C | A | 0.143 | Yes |
| GABRA2 (rs279858) | 0.021 | 0.314 | 98.7 | T | C | 0.493 | Yes |
| HES7 (rs1442849) | 0.596 | 1.000 | 100.0 | C | T | 0.312 | Yes |
| KAT2B (rs9829896) | 0.481 | 1.000 | 100.0 | C | A | 0.610 | Yes |
| NRG1 (rs17664708) | 0.530 | 1.000 | 100.0 | C | T | 0.097 | Yes |
| OPRM1 (rs1799971) | 1.000 | 1.000 | 100.0 | A | G | 0.195 | Yes |
| OPRM1 (rs510769) | 0.535 | 1.000 | 100.0 | C | T | 0.240 | Yes |
| P2RX7 (rs7958311) | 0.306 | 1.000 | 74.0 | G | A | 0.263 | No |

Multiple testing corrections were performed for adjusting multilocus analyses by Bonferroni correction with an effective number of 15 variants; Criteria for variant exclusion: 1) genotyping call rate inferior to 95%; 2) statistically significant departures from HWE (after multiple testing correction); 3) Minor allele frequency (MAF) inferior to 5%; 4) more than one mutant allele; 5) Alleles are based on the NCBI nomenclature.
